# Use of Deep Learning to Develop and Analyze Computational Hematoxylin and Eosin Staining of Prostate Core Biopsy Images for Tumor Diagnosis

**DOI:** 10.1001/jamanetworkopen.2020.5111

**Published:** 2020-05-20

**Authors:** Aman Rana, Alarice Lowe, Marie Lithgow, Katharine Horback, Tyler Janovitz, Annacarolina Da Silva, Harrison Tsai, Vignesh Shanmugam, Akram Bayat, Pratik Shah

**Affiliations:** 1 Program in Media Arts and Sciences, Massachusetts Institute of Technology, Cambridge; 2 Harvard Medical School, Brigham and Women’s Hospital, Boston, Massachusetts; 3 Department of Pathology, Stanford University Medical Center, Stanford, California; 4 Boston University School of Medicine, VA Boston Healthcare, West Roxbury, Massachusetts

## Abstract

**Importance:**

Histopathological diagnoses of tumors from tissue biopsy after hematoxylin and eosin (H&E) dye staining is the criterion standard for oncological care, but H&E staining requires trained operators, dyes and reagents, and precious tissue samples that cannot be reused.

**Objectives:**

To use deep learning algorithms to develop models that perform accurate computational H&E staining of native nonstained prostate core biopsy images and to develop methods for interpretation of H&E staining deep learning models and analysis of computationally stained images by computer vision and clinical approaches.

**Design, Setting, and Participants:**

This cross-sectional study used hundreds of thousands of native nonstained RGB (red, green, and blue channel) whole slide image (WSI) patches of prostate core tissue biopsies obtained from excess tissue material from prostate core biopsies performed in the course of routine clinical care between January 7, 2014, and January 7, 2017, at Brigham and Women’s Hospital, Boston, Massachusetts. Biopsies were registered with their H&E-stained versions. Conditional generative adversarial neural networks (cGANs) that automate conversion of native nonstained RGB WSI to computational H&E-stained images were then trained. Deidentified whole slide images of prostate core biopsy and medical record data were transferred to Massachusetts Institute of Technology, Cambridge, for computational research. Results were shared with physicians for clinical evaluations. Data were analyzed from July 2018 to February 2019.

**Main Outcomes and Measures:**

Methods for detailed computer vision image analytics, visualization of trained cGAN model outputs, and clinical evaluation of virtually stained images were developed. The main outcome was interpretable deep learning models and computational H&E-stained images that achieved high performance in these metrics.

**Results:**

Among 38 patients who provided samples, single core biopsy images were extracted from each whole slide, resulting in 102 individual nonstained and H&E dye–stained image pairs that were compared with matched computationally stained and unstained images. Calculations showed high similarities between computationally and H&E dye–stained images, with a mean (SD) structural similarity index (SSIM) of 0.902 (0.026), Pearson correlation coefficient (PCC) of 0.962 (0.096), and peak signal to noise ratio (PSNR) of 22.821 (1.232) dB. A second cGAN performed accurate computational destaining of H&E-stained images back to their native nonstained form, with a mean (SD) SSIM of 0.900 (0.030), PCC of 0.963 (0.011), and PSNR of 25.646 (1.943) dB compared with native nonstained images. A single blind prospective study computed approximately 95% pixel-by-pixel overlap among prostate tumor annotations provided by 5 board certified pathologists on computationally stained images, compared with those on H&E dye–stained images. This study also used the first visualization and explanation of neural network kernel activation maps during H&E staining and destaining of RGB images by cGANs. High similarities between kernel activation maps of computationally and H&E-stained images (mean-squared errors <0.0005) provide additional mathematical and mechanistic validation of the staining system.

**Conclusions and Relevance:**

These findings suggest that computational H&E staining of native unlabeled RGB images of prostate core biopsy could reproduce Gleason grade tumor signatures that were easily assessed and validated by clinicians. Methods for benchmarking, visualization, and clinical validation of deep learning models and virtually H&E-stained images communicated in this study have wide applications in clinical informatics and oncology research. Clinical researchers may use these systems for early indications of possible abnormalities in native nonstained tissue biopsies prior to histopathological workflows.

## Key Points

**Question:**

Can deep learning systems perform hematoxylin and eosin (H&E) staining and destaining and are the virtual core biopsy samples generated by them as valid and interpretable as their real-life unstained and H&E dye–stained counterparts?

**Findings:**

In this cross-sectional study, deep learning models were trained using nonstained prostate core biopsy images to generate computationally H&E stained images, and core biopsy images were extracted from each whole slide, consisting of approximately 87 000 registered patch pairs of 1024 × 1024 × 3 pixels each. Comprehensive analyses of virtually stained images vs H&E dye–stained images confirmed successful computational staining.

**Meaning:**

The findings of this study suggest that whole slide nonstained microscopic images of prostate core biopsy, instead of tissue samples, could be integrated with deep learning algorithms to perform computational H&E staining and destaining for rapid and accurate tumor diagnosis.

## Teaser

This cross-sectional study describes the development of deep learning algorithms to virtually dye prostate tissue samples and evaluates the effectiveness of images generated using this method vs hematoxylin and eosin–stained images for histopathological diagnosis of tumors.

## Introduction

Cancer is the second leading cause of death in the US.^1^ An estimated 164 690 US men were diagnosed with prostate cancer and 29 430 died of the disease in 2018.^1^ While the survival rate for people with localized prostate cancer is more than 98%, it is reduced to 30% when the cancer spreads to other parts of the body, such as distant lymph nodes, bones, or other organs.^1^ This reduction in survival rate may be prevented with early diagnosis. The current criterion standard for prostate cancer diagnosis uses dye staining of core biopsy tissue and subsequent microscopic histopathologic examination by trained pathologists.^2^ Hematoxylin and eosin (H&E) is the most widely used dye staining method that leverages interactions of H&E dyes with tissues for visualization.^3^ Every day, up to 3 million slides are stained with this technique. The total end-to-end processing time from slide scanning to automated staining is less than 10 minutes. However, tissue processing of H&E dye staining for paraffin sections can take between 7 to 26 hours.^4^ Microscopic diagnosis of tumors using H&E dye–stained biopsy slides presents challenges, such as inconsistencies introduced during tissue preparation and staining and human errors, and it also requires significant processing time, imaging systems, and procedural costs.^5^ Other key challenges include sampling time, which can limit the amount of tissue that can be stained owing to time and cost involved, resulting in evaluation of only three 4-μm sections of tissue to represent a 1-mm diameter core.^6^ Irreversible dye staining of tissues leads to loss of precious biopsy samples that are no longer available for biomarker testing. Automated, low-cost, and rapid generative algorithms and methods that can convert native nonstained whole slide images (WSIs) to computationally H&E stained versions with high precision could be transformative by benefiting patients and physicians and by reducing errors and costs.

Whole-slide pathological images are approved by the US Food and Drug Administration^7^ for cancer diagnosis and can rapidly be integrated into machine learning and artificial intelligence algorithms for automatic detection of cellular and morphological structures to tumors and virtual staining.^8^ Studies testing operational feasibility and validation of results obtained by generative models and machine learning algorithms in controlled clinical trials or hospital studies for virtual staining of whole-slide pathology images do not exist, to our knowledge, precluding clinical deployment of these systems.

**Figure 1.**
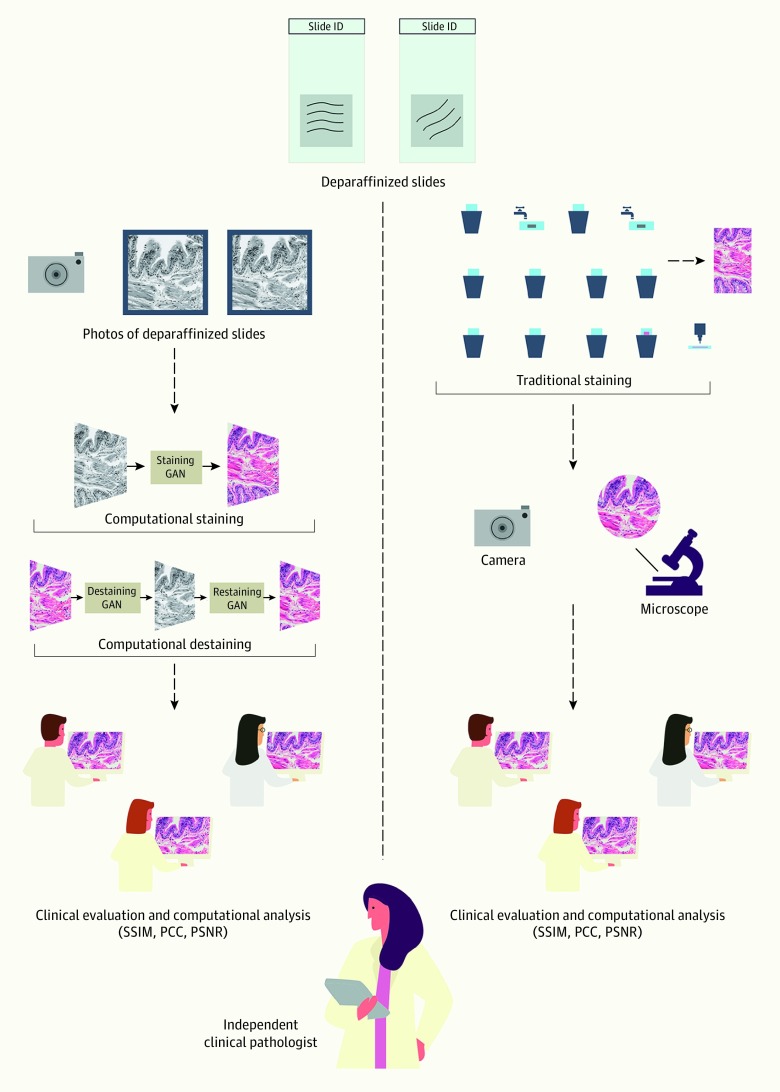
Overview of the Staining Processes Left, Computational staining and destaining of whole slide prostate core biopsy images with conditional generative adversarial neural networks (CGAN). Right, traditional staining with hematoxylin and eosin (H&E) dyes using physical prostate core tissue biopsy slides. PCC indicates Pearson correlation coeffecient; PSNR, peak signal to noise ratio; and SSIM, structural similarity index.

We previously communicated convolutional neural networks for learning associations between expert annotations of disease and fluorescent biomarkers manifested on RGB (red, green, and blue channel) images and their complementary nonfluorescent pixels found on standard white-light images.^9^ Subsequently, we communicated conditional generative adversarial neural networks (cGANs) that accept native nonstained prostate core biopsy autofluorescence RGB WSIs and computationally stain them in a manner visually similar to H&E by learning hierarchical nonlinear mappings between image pairs before and after H&E dye staining.^10^ In this study, we report several novel mechanistic insights and methods to facilitate benchmarking and clinical and regulatory evaluations of computationally H&E stained images for oncological applications. Specifically, we trained high fidelity, explainable, and automated computational staining and destaining algorithms to learn mappings between naturally autofluorescent pixels^11^ of nonstained cellular organelles and their stained counterparts. We also devised robust loss function for our machine learning algorithms to preserve tissue structure. Furthermore, we established that our virtual staining neural network models were generalizable to accurately stain previously unseen images acquired from patients and tumor grades not part of training data. We generated neural activation maps to provide the first instance of explainability and mechanisms used by cGANs models for virtual H&E staining and destaining and establish computer vision analytics to benchmark the quality of generated images. Finally, we evaluated computationally stained images for prostate tumor diagnoses with multiple pathologists for clinical evaluation (Figure 1).

By describing explainable algorithms and quantitative methods that can consistently, rapidly, and accurately perform computational staining and destaining of prostate biopsy RGB WSI, this study communicates a detailed method and process that may be useful to generate evidence for clinical and regulatory authentication of computationally H&E stained images. However, greater numbers of virtually stained H&E images sourced from larger pools of patients are needed for prospective evaluation of such models.

## Methods

Partners Human Research Committee approved our study protocol for utilization of excess material from prostate core biopsies performed in the course of routine clinical care between January 7, 2014, and January 7, 2017, at Brigham and Women’s Hospital, Boston, Massachusetts. Informed consent was waived because data were deidentified and samples were obtained as part of routine clinical care. Deidentified WSIs and electronic health record (EHR) data were transferred to the Massachusetts Institute of Technology for processing and analyses and was exempt from institutional review board review per the Massachusetts Institute of Technology’s Committee on the Use of Humans as Experimental Subjects. This study is reported following Transparent Reporting of a Multivariable Prediction Model for Individual Prognosis or Diagnosis (TRIPOD) reporting guideline.

### Study Population

Thirty-eight men (mean [SD] age, 66.2 [8.9] years), including 32 white men (84%), 4 African American men (11%), 1 Hispanic/Latino man (3%), and 1 Dominican man (3%), provided 46 core biopsy samples (eTable 1 in the Supplement). Each biopsy sample contained 1 to 6 cores of tissue, and 0% to 100% of each tissue core contained prostatic adenocarcinoma of various Gleason grades. Individual prostate tissue needle core biopsy images from each whole slide image were extracted, which resulted in 102 high-resolution native nonstained and H&E dye–stained image pairs that were registered to form 102 RGB WSIs pair images.

Deparaffinized nonstained slides were scanned at 20 × magnification. Subsequently, slides were stained with H&E dye on a Dako autostainer (Agilent), and these stained slides were rescanned at 20 × magnification at Harvard Medical School Tissue Microarray and Imaging Core. Deidentified data in the form of nonstained and H&E dye–stained images at 20 × magnification were analyzed at Massachusetts Institute of Technology. Individual prostate tissue needle core biopsy images from each WSI were extracted, which resulted in high-resolution native nonstained and H&E dye–stained image pairs, which then were registered to form RGB WSI pair images. The RGB WSIs were too large to enter into deep learning networks; therefore, each image was cropped into multiple patches of 1024 × 1024 × 3 pixels, resulting in approximately 87 000 registered pair patches.

### Training and Validation Data Sets

The numbers of image patches and their Gleason grade tumor grading used in this study for test data were 7019 Gleason grade 3 tumors, 6149 Gleason grade 4 tumors, and 270 Gleason grade 5 tumors (eTable 1 in the Supplement) and were sufficient to study the computational staining problem. Core biopsy images from a larger and diverse patient population from additional medical centers are currently being procured to improve generalizability of clinical findings reported in this study. The registered data set of images (WSI pairs) was divided into approximately 74 000 training and 13 500 validation image patches. Validation and training data sets were balanced to include images from healthy patients as well as patients with different grades of prostate tumors and of each tumor grade. More information about the data collection process and training and validation sample descriptions can be found in eAppendix 1 and eTable 1 in the Supplement.

### Machine Learning Model Architecture and Training

A cGAN pix2pix-based model was trained to learn distribution and mappings among registered images in the training data set.^12^ The staining model accepts a native nonstained RGB WSI and generates computationally H&E stained RGB WSI. The destaining model reverses the process and generates computationally destained images from H&E dye–stained RGB WSI patches. A novel Pearson correlation coefficient (PCC) term was added to the cGAN loss function to improve the quality, enforce tissue structure preservation of computationally stained images, and help reduce the tiling artifacts in the computationally stained image. More details about loss function and technical implementation are presented in eAppendix 2 in the Supplement.

### Image Evaluation Metrics

The computationally stained image patches generated by our model were compared with H&E dye patches to obtain a quantitative measure of the generated images. We used PCC, peak signal to noise ratio^13^ (PSNR) and structural similarity index^14^ (SSIM) to quantify similarities and differences between a given pair of images at a pixel level. The values of PCC and SSIM range from 0 to 1, with higher values indicating higher levels of similarity. Accurate values of PSNR for wireless transmission quality loss are considered to be between 20 dB to 25 dB,^15,16^ and higher PSNR is better. The mean and total increase in pixel intensity after computationally staining and destaining were calculated by subtracting the mean pixel intensity of the second image from the first.

### Clinical Validation of Computationally H&E Stained RGB WSI

Computationally stained patches from the test data set were used to reconstruct 13 RGB WSIs. Their corresponding RGB WSI H&E dye–stained images were used as ground truth examples and also labeled for tumors. A single-blind study was conducted for evaluation of all images for prostate cancer diagnosis. Four board-certified and trained expert pathologists provided detailed labels in the form of free-form outlines encompassing tumors, indicating tumor regions (with grade) and other atypical manifestations on computationally stained and corresponding H&E dye–stained images. In the first round, 2 randomly selected pathologists were provided computationally stained images while H&E dye–stained images were given to the other 2 raters. After a period of 4 weeks, the image sets were swapped among the pathologists, and another round of annotations were conducted. Pathologists annotated images in the form of free-hand drawing using the Sedeen Viewer (PathCore) on identical notebook computer screens (Dell Computers). By using different colors corresponding to each tumor grade, annotations were classified with tumor grade: Gleason grade 3, Gleason grade 4, or Gleason grade 5. A separate comments box was used to note other clinical observations and for anatomical features. The annotations and the associated labels (ie, Gleason grades 3, 4, or 5) were extracted from the XML files generated by Sedeen using the labels and annotations using Python code. The agreement was calculated in the form of intersection over union which measures the number of pixels on computationally stained and corresponding H&E dye–stained images that have common raters annotations divided by the total number of pixels present across both images.^17^ An independent fifth clinical pathologist ratified corresponding computationally stained images. Accuracies and errors were calculated using pixel-by-pixel overlap in the labels. Color-coded error overlaid validation images were generated visualizing the true positives, false positives, and false negatives (eFigure 1 in the Supplement).

### Activation Maps

Input images containing Gleason grade 3, 4, and 5 signatures were entered into our trained computational staining network to visualize activation maps for each input image. Full-scale RGB WSIs at 20 × resolution were collected and constructed for 8 image data sets (ie, 4 pairs), each with 13 images: ground native nonstained, ground H&E dye–stained, predicted reconstructed computationally stained, and predicted reconstructed destained (also referred to as *predicted destained images*) (eAppendix 3 in the Supplement). A total of 448 unique patches in each of the 8 data sets with no overlap were created for each data set and set to size 1024 × 1024 × 3 (3 color channels). For each matching patch pair to be entered into computational staining or destaining models, we examined the grid linearly and isolated consolidated activation maps from layer 1 to layer 19 (eFigure 2 in the Supplement). These individual activation maps concatenated together to form a single image per layer of the model architecture. Examples of concatenated activation maps are presented in eFigure 3 and eFigure 4 in the Supplement. The normalized mean-square error (MSE) was calculated for all layers between the matching patch pairs (eAppendix 3 and eFigure 5 in the Supplement).

## Results

### Quantitative Evaluation of Computationally Stained and Destained Images

Computationally H&E stained WSIs were compared pixel-by-pixel to corresponding H&E dye–stained images (Table 1). We calculated SSIM and PSNR (in logarithmic dB), and PCC were used as quality measures of computationally stained images with H&E dye–stained images regarded as ground truth.^18,19^ A mean (SD) SSIM of 0.902 (0.026), PCC of 0.962 (0.096), and PSNR of 22.821 (1.232) dB were calculated, indicating high accuracy of computational H&E staining of test images. High PCC accuracy scores (81.8% of patches with PCC ≥0.7 and 39.4% patches with PCC ≥0.8) indicate that computationally stained patches matched H&E dye–stained patches at a pixel level.

**Table 1.** Comparison of Computationally Stained and Ground Truth H&E Dye–Stained Images, and of Computationally Destained vs Ground Truth Native Nonstained Images

Image	Computationally stained vs H&E stained			Computationally destained vs nonstained		
	PCC, %^a^	SSIM, %^b^	PSNR, db^c^	PCC, %^a^	SSIM, %^b^	PSNR, db^c^
1	95.0	86.0	20.563	95.1	85.3	23.486
2	95.2	89.1	22.387	96.5	89.5	25.706
3	94.9	86.0	20.683	96.4	86.6	24.871
4	95.7	92.9	22.870	96.8	93.6	27.469
5	96.0	94.7	24.838	97.0	94.9	21.194
6	95.5	91.4	22.903	95.7	91.4	25.285
7	96.0	88.1	22.486	93.8	86.5	21.863
8	96.8	93.1	24.132	95.9	92.7	26.164
9	97.8	89.0	23.411	96.8	87.4	24.165
10	95.6	91.3	23.177	96.7	92.7	27.359
11	97.2	90.7	23.945	98.4	89.9	26.792
12	97.5	90.2	23.200	96.3	89.9	24.957
13	96.5	89.9	22.074	97.0	90.0	26.082
Total, mean (SD)	96.1 (1.0)	90.2 (2.6)	22.821 (1.232)	96.3 (1.1)	90.0 (3.0)	25.646 (1.943)

Abbreviations: H&E hematoxylin and eosin; PCC, Pearson correlation coefficient; PSNR, peak signal to noise ratio; SSIM, structural similarity index.

^a^ Pearson correlation coefficient of 1.0 indicates a perfect match.

^b^ Structural similarity index of 1.0 indicates perfect match.

^c^ Peak signal to noise ratio of 22 dB or more is considered high quality.

Comparison of each RGB color channel’s pixel intensities (PXI) between native nonstained and computationally stained images (−42 PXI), and those between native nonstained and H&E dye–stained images (−44 PXI) show that computationally stained images had mean intensity difference of only 2 PXI (eTable 2 in the Supplement). Similar low differences between ground truth H&E dye and computationally stained images were observed after comparing individual color channels listed in eTable 3 in the Supplement: red channel (unstained vs computationally stained: −58 PXI; unstained vs H&E stained: −58 PXI; H&E vs computationally stained: 0 PXI), green channel (unstained vs computationally stained: 6 PXI, unstained vs H&E stained: −8 PXI, H&E vs computationally stained: 2 PXI), and blue channel (unstained vs computationally stained: −62 PXI, unstained vs H&E stained: −65 PXI, H&E vs computationally stained: 3 PXI).

Prostate core biopsy H&E dye–stained images were computationally destained and compared with native nonstained images. Mean (SD) PCC was 0.900 (0.030), mean (SD) SSIM was 0.963 (0.011), and mean (SD) PSNR was 25.646 (1.1943) dB (Table 1), thus showing high similarities with native ground truth nonstained images. RGB pixel intensities between computationally destained and H&E dye–stained images (47 PXI), and native nonstained and H&E dye–stained images (44 PXI) also indicated that computationally destained and ground truth nonstained images only had 3 PXI difference in their overall intensities (eTable 2 in the Supplement). These results indicate high fidelity of learning, reproducing, and erasing of multichromatic information by computational H&E staining and destaining algorithms. Mean (SD) change in pixel intensities in the red and blue channels were higher compared with the green channel likely because H&E dye predominantly consists of blue and red or pink colors.

### Analyses of Physician Annotations

Intersection over union indicating agreements or disagreements among pathologists examining the same set of images (intra–intersection over union) was calculated by pixel-by-pixel comparisons of their tumor and nontumor annotations (eTable 4 in the Supplement). Pathologists examining H&E dye–stained images had high mean (SD) intra–intersection over union agreement scores for diagnosing any tumors (0.81 [0.07]). Pathologists examining computationally H&E stained images also had high and comparable mean (SD) intra–intersection over union agreement scores for diagnosing any tumor (0.77 [0.08]). These results indicated high internal consistency in clinical diagnoses provided by each set of pathologists on their respective images. Furthermore, tumor diagnoses using computationally stained images were not associated with rater’s sensitivity or specificity while detecting tumors.

Tumor labels provided by 2 sets of physicians in our single-blind study on ground truth H&E dye–stained images vs computationally stained images were then compared using inter–intersection over union agreement score metric^17^ (Table 2). An overall inter–intersection over union score of 0.79 was calculated for any tumor diagnoses. The mean (SD) inter–intersection over union agreement score for Gleason grade 3 tumors was 0.70 (0.17) and 0.73 (0.15) for Gleason grade 4 labels. Gleason grade 5 tumors are rare, and we only had 1 example in validation data that was annotated, with an accuracy of 0.64 (Table 2). The mean (SD) inter–intersection over union agreement score for annotations of healthy areas in the tissue where no tumors were found on images was 0.90 (0.12) (Table 2). These results indicate that our trained machine learning models can accurately generate both tumor and nontumor signatures via computational H&E staining. Physician raters showed concordance and comparable sensitivity and specificity in diagnosis made using H&E dye–stained images compared with those made by using computationally stained images.

**Table 2.** Intersection Over Union–Based Agreement Among Pathologists for Tumor Signatures Provided Using Computationally Stained Images Compared With Pathologists Using Ground Truth Hematoxylin And Eosin Dye–Stained Images

Image	Intersection over union^a^				
	Any tumor	Healthy	Gleason grade		
			3	4	5
1	0.90	0.96	0.90	NA	NA
2	0.86	0.55	NA	0.78	NA
3	NA	1.00	NA	NA	NA
4	0.92	0.89	0.76	NA	NA
5	0.52	0.90	NA	0.49	0.64
6	0.80	0.93	0.58	NA	NA
7	0.70	0.94	0.53	NA	NA
8	0.79	0.92	NA	0.77	NA
9	0.58	0.96	0.48	NA	NA
10	0.86	0.86	0.70	0.72	NA
11	0.92	0.99	0.92	NA	NA
12	NA	1.00	NA	NA	NA
13	0.93	0.78	NA	0.89	NA
Mean	0.79 (0.14)	0.90 (0.12)	0.70 (0.17)	0.73 (0.15)	0.64 (0)

Abbreviation: NA, not applicable

^a^ Higher intersection over union score is better, with a score of 1.0 indicating perfect match of labels.

### Clinical Evaluations of Computationally Stained Images

**Figure 2.**
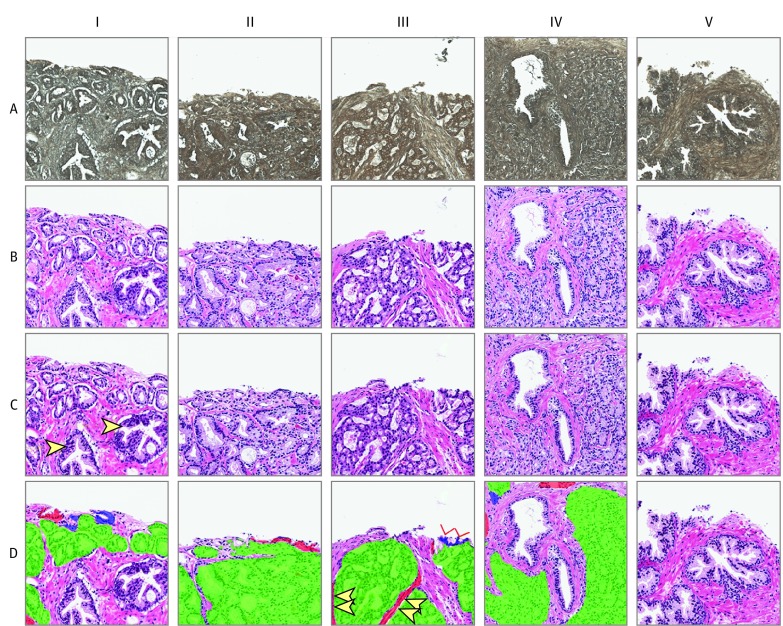
Representative Image Patches Generated by the Computational Staining Neural Network and Their Comparison With Corresponding Ground Truth Hematoxylin and Eosin (H&E) Dye–Stained Images Row A, Deparaffinized native nonstained image patches entered into the neural network. Row B, Ground truth H&E dye–stained patches. Row C, computationally H&E stained patches generated by the neural network. Arrows in C-I indicate the 2 benign glands, all other glands represent tumors. Row D, shows computationally H&E stained patches overlaid with colors indicating agreements and disagreements between physician annotations on these images compared with ground truth H&E dye–stained images. Variation in labeling detail by annotators (arrows) are shown in D-III. Green indicates true positive; blue, false negative; and red, false positive.

Figure 2 shows representative input nonstained image patches that had Gleason grade 3 or 4 tumors or were benign and their computational H&E staining and accuracy calculated using annotations by multiple physicians. It is evident that the computationally H&E stained patches represent tumor signatures with high accuracy, and pathologists were able to correctly identify tumors. Most observed disagreements between raters did not represent misidentification of glands as benign or malignant. Instead, they showed differences in rater annotation at borders of tumor labels, mainly due to differences in labeling style. eAppendix 4 and eFigure 1 in the Supplement provide detailed clinical evaluations and outcomes of individual patches and reconstructed RGB WSI computationally H&E stained images.

### Comparison With Patient Records

Most of the diagnoses rendered using computationally stained images agreed with the corresponding initial clinical diagnosis reported in electronic health records (EHRs) (eTable 5 in the Supplement), supporting the validity of the generated images for tumor detection and diagnoses. Most of the samples showed identical tumor fractions and Gleason grading. None of the differences between EHRs and diagnoses based on computationally H&E stained images were clinically significant with regard to treatment decisions (eAppendix 5 in the Supplement). We were able to overturn originally reported results in patient records in 2 instances using computationally stained images (eFigure 1 and eAppendix 5 in the Supplement).

### Analysis and Explanation of Neural Network Activation Maps

Neural activation maps of trained staining and destaining cGAN models were analyzed after entering healthy or Gleason grade 3, 4, or 5 images patches (Figure 2). In this study, we did not use a classification approach to identify image features, but rather performed pixel-by-pixel visualization, explanation, and intensity ranking (>200 value) of various cGAN kernels to create an activation map of a particular nonstained image patch (healthy vs with a particular Gleason tumor grade) as it passes through each network layer while getting stained (eFigure 2 in the Supplement).

**Figure 3.**
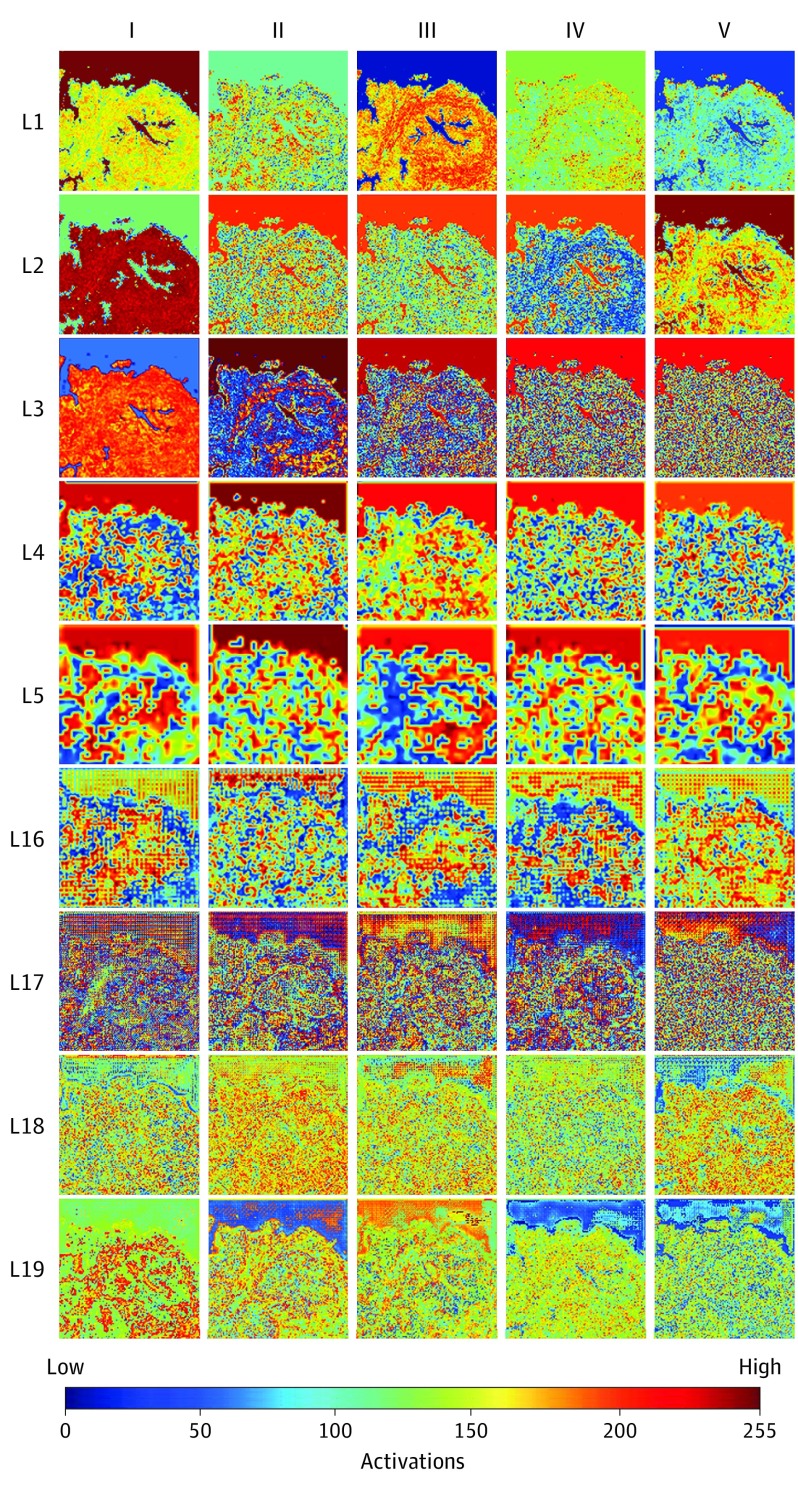
Activation Maps of Kernels of Trained Generator Neural Network Model Activation maps of kernels of trained generator neural network model layers after feeding a native nonstained prostate core biopsy image patch without tumor as it gets computationally hematoxylin and eosin–stained. Rows show top 5 activation maps from layers L1 to L5 and L16 to L19 arranged in decreasing order of their activations from left to right (columns I-V).

We demonstrate and compare presence of unique low- and high-level features in input images that activate neurons and feature maps in the cGAN generator network (Figure 3; eFigure 3 and eFigure 4 in the Supplement). For example, initial layers of the convolutional layers in the generator detected low-level features, such as tissue geometry, edges, corners, shapes, and a few changes in color (Figure 3; eFigure 3, and eFigure 4 in the Supplement). We observed well-demarcated boundaries between tissues and background and gross distinctions between glands and stroma are suggested (Figure 3) or are well defined (eFigure 3 in the Supplement). Kernels of initial layers of trained models thus help with differentiating tissue from background and morphological tasks to define higher order anatomical structures. The later convolutional layers leverage previously learned low level features and ability to differentiate tissue from background with fine-grained structures, such as anatomical arrangement of nuclei and tumor signatures (Figure 3; eFigure 3 and eFigure 4 in the Supplement). eFigure 4 in the Supplement shows additional examples of indeterminate atypical glands and tumors with edge or crush artifacts that are well preserved on the computationally generated images but were differentially designated as tumor or nontumor by raters. The activation maps of kernels of various generator neural network layers after feeding H&E dye–stained patch with Gleason grade 4 and 5 prostate tumors were demonstrated.

We compared kernel activation maps of all 448 validation image patches used to test our trained staining and destaining machine learning models with corresponding ground truth dye-stained and native nonstained images (eFigure 5 in the Supplement). The MSE was calculated by comparing activation maps generated by each of the 19 neural network layers in response to pairs of images being evaluated. The MSE was low for the first layer, increases for second layer, and then decreases for the remaining layers. These results, in unification with our detailed SSIM, PSNR, PCC, and physician validation, provide significant evidence of the high quality of computationally stained and destained images, with consequent high sensitivity and specificity in diagnosing tumors using them.

## Discussion

Most surgical and medical treatments for cancer, including chemotherapy, endocrine therapy, and immunotherapy, are dictated by histopathologic examination and diagnosis. Increase in use of core biopsies for diagnosis, in place of larger surgical biopsies, has resulted in significant decrease in the volume of tumor available for performing an ever-increasing battery of biomarker testing for diagnostic, prognostic, and predictive information. In this cross-sectional study, computationally stained and destained images were evaluated by multiple image analytics and matched ground truth images with high similarity. The high quality of the computationally stained and destained images were comprehensively and stringently validated using pixel-based comparison (eg, MSE, PSNR), spatial structural comparison (eg, SSIM), and localized correlational comparison (eg, PCC), which revealed their macroscopic and microscopic suitability for clinical deployment.

Evaluation by trained pathologists showed tumorous and healthy tissues were morphologically well represented most of the computationally stained images with high accuracy. The glands and stroma of benign prostatic tissue and carcinoma were identifiable, showing preserved architectural features (ie, location and shape of the glands), defined gland/stromal interface, and cytological characteristics (ie, location and appearance of the nuclei and nucleoli, if present). Most of the differences in annotations were observed either on the tumor/nontumor interface or boundary or the biopsy boundary. This can be attributed to the labeling style of individual raters.^17^ Previous studies have reported that human readers show substantial variability and lower performance compared with computer algorithms in terms of tumor segmentations.^8,20^ A similar limitation of using a human reader panel to establish a reference standard for evaluation of computer algorithms may have affected this study. In validation images, presence of morphologically ambiguous glands, a known histopathological dilemma that clinically requires additional testing for confident diagnosis, also led to differing labels among raters, as they were asked to categorize each gland as benign or malignant without assistance from supplemental studies. In most cases, these ambiguous cases were well represented in the computationally stained images but led to labeling differences owing to the ambiguity of these regions of interest. Small difference calculated by PSNR, SSIM, and PCC, independent of the human raters, may also be in part due to registration differences in small, out-of-focus areas during whole-slide imaging. Input image pairs (ie, nonstained and H&E stained) used for training in our work were corrected for differences in field of view, illumination, and focal planes, but they may still have had minor variances. However, these small variances in computationally stained images had no effects on overall clinical assessments. Color variations in digital slides may have been due to differences in staining reagents, thickness of tissue sections, and staining protocols, which can negatively affect clinical diagnoses. We report minimal color variation across our computationally stained H&E images, as seen by their uniform overall RGB and individual RGB channel intensity values, which often matched training images. Physician raters in the study did not report difficulty in reading colors of nuclei, glands, cells, and tumors in computationally stained images, which was ratified by an additional independent pathologist. Thus, the trained neural network model reproduces a consistent and normalized color hue from the vast training data set that does not affect clinical decision-making from computational images. The subsequent absence of false-positive errors in healthy tissue cores of patients illustrates the fine grain reproduction of our computationally stained and destained images. We were also pleased to find high concordances between diagnoses made using the computationally stained images in this study and the patient’s EHR. In fact, we found 2 instances in which the diagnoses made using computationally stained images overturned the initial findings in the EHR. In both cases, additional laboratory tests and clinical examinations were performed to confirm our findings. These results demonstrated that raters and the tumor diagnosis performed using computationally stained WSI used in our study matched or exceeded the initial microscopic diagnosis performed using H&E-stained tissue slides after prostate biopsy extraction.

Virtual staining of histopathological slide images has been reported using approaches with signals that require long detection times,^21^ dye staining of nonstained specimens prior to imaging,^22^ laser illumination and excitation with specific wavelengths,^23^ sparse sampling, and poor depth resolution.^22,24^ Previous virtual staining studies have performed limited analytics^25^ to benchmark the quality of their virtually stained images. Most previous studies did not perform pixel-level comparisons with ground truth images and used small numbers of nonblinded raters who used coarse annotations without tumor gradations.^26,27^ While other studies have reported no clinical validation and benchmarking of their results.^28,29,30^ Similarly, previous deep learning research for virtual staining has used specialized illumination sources and did not report robust validation studies on mechanisms to establish computer vision or diagnostic utility of generated images.^8^ Bayramoglu et al^31^ virtually stained lung tissue slides multispectral images with a cGAN and achieved an SSIM of 0.3873, but they performed no clinical validation. Bulingame et al^32^ used cGAN to convert H&E-stained pancreas slide RGB images to immunofluorescence images and achieve an SSIM of 0.883, but they also did not report clinical validation of generated images. Two studies by Rivenson et al^33,34^ used a fluorescence scope with specialized ultraviolet filters to capture various tissue biopsy images and virtually H&E stain them using a neural network. Results and findings communicated in our study differ from previous deep learning based virtual staining studies in several key aspects. As examples, a wide field fluorescence microscope to image tissue^33,34^ vs the nonfluorescent mode of the Food and Drug Administration–approved and widely available automated slide scanning system to capture images used in our study. A single pathologist compared anatomical features among virtually stained images using coarse labels, and pixel-level comparisons between tumor labels on virtual and ground truth images or concordance with EHR of patients were not conducted to calculate true- and false-positive occurrences of tumor diagnoses reported in that study.^33,34^ Computational destaining of tissue images and stringent image analytics, such as PSNR or PCC, to benchmark quality of virtually stained images have not been reported in previous deep learning based studies.^31,32,33,34^ Analysis or visualization of key neural network kernels and image features that get activated during the staining process have not been investigated, thus precluding mechanistic insights or mathematical validation of previous findings reported in literature.^31,32,33,34^

In this study, we evaluated trained neural network models that computationally stained native unlabeled RGB images of prostate core biopsy (acquired without band pass filters or specialized hardware) with anatomical features of prostate and reproduce cancer tumor signatures with high accuracies. Computational pixel-by-pixel analysis and comparisons using PSNR, SSIM, and PCC demonstrated high similarities between our computationally stained images and their H&E dye–stained counterparts. Pixel-by-pixel changes in RGB color channels after computational staining and destaining by neural networks matched corresponding changes in RGB intensity when native nonstained images were H&E dye–stained in pathology laboratories vice versa. Detailed clinical validation in a single blind study found high interrater and intrarater agreements, calculated by pixel-by-pixel analyses of tumor labels provided by multiple board certified and trained physicians. Computationally stained images thus accurately represented healthy tissue as well as tumors of different Gleason grades, which were easily detected by human visual perception. Clinical diagnoses made using computationally stained images in our study were consistent with tumor diagnoses reported in EHRs. We investigated layers of generator neural networks and calculated activation of kernels during staining of different prostate tumor grades and benign tissue signatures to visualize and explain the process of computational H&E staining and destaining. Activation maps of our trained neural network models during computational staining or destaining of test images were highly similar to H&E dye–stained or native nonstained images. Thus, by visualizing and comparing activation feature maps of kernels of trained models, this work also presents the first explainable deep neural network framework for computationally H&E staining or destaining of native RGB images, to our knowledge.

### Limitations

There were a few limitations of this study. The validation process for tumor diagnoses and Gleason grading of computationally H&E stained images can be affected by interobserver variability. For example, despite using a large rater panel in a single-blind study, tumor regions annotated by pathologists on WSIs are often coarse and may contain nonrelevant tissue that increases disagreements. Additional fine-grained image annotation tools are needed for precise validation of results generated by computational staining algorithms. The amount of data or number of patients used in this study was not exhaustive for clinical trials or other regulatory evaluations. The numbers of images used were found to be sufficient for accurate H&E staining, and adding additional images could result in modest improvements. This study described detailed methods that could be used to interpret deep learning systems and virtually H&E-stained images derived from them by computer vision analytics, and our findings may be useful to clinical and regulatory science researchers in the field. Because Gleason grade 5 tumors are quite rare, only 1 WSI was evaluated in the validation data that was annotated with intersection over union accuracy of 0.64. More nuanced diagnostic validation requiring evaluation of tissue beyond typical H&E staining (eg, evaluation requiring immunohistochemical staining) is not addressed in this study and is a possible development area of this work. The clinical outcomes from this study are limited to the evaluation of prostate core biopsies as a representative tissue type, but our methods and approach should generalize to other tissue biopsy evaluations. Application to other tumor types within core biopsies or to resection specimens of prostate cancer or other conditions will be evaluated in future work.

## Conclusions

This cross-sectional study communicates methods and processes that may be useful for additional research and validation of computational H&E staining deep learning models and images generated by them. Adoption of these systems may reduce the time and effort required for manual staining and slide preparation, and more importantly, enable the preservation of precious tissue samples which could be used in a targeted fashion for biomarker evaluation. Greater numbers of virtually stained H&E images sourced from larger pools of patients are needed before prospective clinical evaluation of models described in this study can begin.^35,36,37^

## Notes

Supplement. **eAppendix 1.** Data Collection and Image Registration Process **eTable 1.** Data Distribution **eAppendix 2.** Loss Function **eAppendix 3.** Interpretation **eFigure 1.** Color Coded Overlaid Validation Images **eFigure 2.** Visualization and Explanation of Computational Hematoxylin and Eosin Staining Process by Custom Autoencoder Neural Network **eFigure 3.** Activation Maps of Kernels of Trained Generator Neural Network Model Layers **eFigure 4.** Activation Maps of Kernels of Various Generator Neural Network Layers After Entering Hematoxylin And Eosin Dye–Stained Patch With Gleason Grades 4 And 5 Prostate Tumor **eFigure 5.** Comparison of Mean Squared Errors Between Kernel Activation Maps of Pairs of 448 Validation Image Patches Generated by the Trained Neural Network Models **eTable 2.** Mean Pixel Intensity Following Computational Staining and Destaining **eTable 3.** Change in Mean Pixel Intensity in Red, Green, and Blue Channels per Image **eTable 4.** Intrarater Agreement Calculated on Dye Stained and Computationally Stained Images **eAppendix 4.** Evaluation of the Activation Maps of Trained Deep Neural Network **eTable 5.** Comparison of Tumor Grades Between Original Expert Microscopic Diagnosis **eAppendix 5.** Comparison With Patient Records **eReferences**
