# Supplementary material for: Use of Deep Learning to Develop and Analyze Computational Hematoxylin and Eosin Staining of Prostate Core Biopsy Images for Tumor Diagnosis
Source: JAMA Netw Open. 2020 May 20;3(5):e205111. doi: 10.1001/jamanetworkopen.2020.5111 (PMC7240356; doi:10.1001/jamanetworkopen.2020.5111)
Supplement: Supplement. — eAppendix 1. Data Collection and Image Registration Process eTable 1. Data Distribution eAppendix 2. Loss Function eAppendix 3. Interpretation eFigure 1. Color Coded Overlaid Validation Images eFigure 2. Visualization and Explanation of Computational Hematoxylin and Eosin Staining Process by Custom Autoencoder Neural Network eFigure 3. Activation Maps of Kernels of Trained Generator Neural Network Model Layers eFigure 4. Activation Maps of Kernels of Various Generator Neural Network Layers After Entering Hematoxylin And Eosin Dye–Stained Patch With Gleason Grades 4 And 5 Prostate Tumor eFigure 5. Comparison of Mean Squared Errors Between Kernel Activation Maps of Pairs of 448 Validation Image Patches Generated by the Trained Neural Network Models eTable 2. Mean Pixel Intensity Following Computational Staining and Destaining eTable 3. Change in Mean Pixel Intensity in Red, Green, and Blue Channels per Image eTable 4. Intrarater Agreement Calculated on Dye Stained and Computationally Stained Images eAppendix 4. Evaluation of the Activation Maps of Trained Deep Neural Network eTable 5. Comparison of Tumor Grades Between Original Expert Microscopic Diagnosis eAppendix 5. Comparison With Patient Records eReferences [file jamanetwopen-3-e205111-s001.pdf]

Supplementary Online Content

Rana A, Lowe A, Lithgow M, et al. Use of deep learning to develop and analyze computational hematoxylin and eosin staining of prostate core biopsy images for tumor diagnosis. *JAMA Netw Open*. 2020;3(5):e205111. doi:10.1001/jamanetworkopen.2020.5111

eAppendix 1. Data Collection and Image Registration Process
eTable 1. Data Distribution
eAppendix 2. Loss Function
eAppendix 3. Interpretation
eFigure 1. Color Coded Overlaid Validation Images
eFigure 2. Visualization and Explanation of Computational Hematoxylin and Eosin Staining Process by Custom Autoencoder Neural Network
eFigure 3. Activation Maps of Kernels of Trained Generator Neural Network Model Layers
eFigure 4. Activation Maps of Kernels of Various Generator Neural Network Layers After Entering Hematoxylin And Eosin Dye–Stained Patch With Gleason Grades 4 And 5 Prostate Tumor
eFigure 5. Comparison of Mean Squared Errors Between Kernel Activation Maps of Pairs of 448 Validation Image Patches Generated by the Trained Neural Network Models
eTable 2. Mean Pixel Intensity Following Computational Staining and Destaining
eTable 3. Change in Mean Pixel Intensity in Red, Green, and Blue Channels per Image
eTable 4. Intrarater Agreement Calculated on Dye Stained and Computationally Stained Images
eAppendix 4. Evaluation of the Activation Maps of Trained Deep Neural Network
eTable 5. Comparison of Tumor Grades Between Original Expert Microscopic Diagnosis
eAppendix 5. Comparison With Patient Records
eReferences

This supplementary material has been provided by the authors to give readers additional information about their work.

© 2020 Rana A et al. *JAMA Network Open.*

eAppendix 1. Data Collection and Image Registration Process

Data collection, transfer and processing of whole slide images: Thirty-eight patients (mean age 66.2 years) consisting of White, African American, Hispanic/Latino, and Asian men provided forty-six core biopsy samples. Of these, nine patients had known prostate cancer diagnosis and were undergoing active surveillance. Eighteen patients underwent subsequent prostatectomy and the remaining were either healthy or undergoing prostate cancer treatment at Brigham and Women's Hospital. Each biopsy sample contained one to six cores of tissue. Zero to 100% of each tissue core contained prostatic adenocarcinoma of various Gleason grades. Samples were enriched for higher-grade tumors (Gleason grade 4 and 5). Forty-six non-stained and corresponding H&E dye stained RWSI were collected from 38 patients and imaged at 20x magnification. Briefly, prostate core biopsy specimens were immediately fixed in 10% formalin, paraffin embedded, cut into 4-micron thick sections and placed on standard glass slides that were placed in archival storage at room temperature. Deparaffinized non-stained slides were scanned with the Aperio ScanScope XT system (Leica Biosystems, Buffalo Grove, IL) at 20x magnification. Subsequently, the slides were stained with H&E dye on the Agilent Dako Autostainer (Agilent, Santa Clara, CA), and these stained slides were re-scanned on the Aperio ScanScope XT at 20x magnification at Harvard Medical School Tissue Microarray & Imaging Core. Deidentified data in the form of non-stained and H&E dye stained images at 20x magnification were analyzed at Massachusetts Institute of Technology. Individual prostate tissue needle core biopsy images from each whole slide image were extracted. Extracted core images were horizontally or vertically rotated to reduce non-tissue pixels. This resulted in 102 high-resolution native non-stained and H&E dye stained image pairs.

Image registration and processing: Deparaffinized single core images (henceforth called as non-stained images) and subsequent H&E dye stained single core images of the same biopsy (henceforth called as H&E dye stained images) were registered using Photoshop CC software (Adobe Systems, San Jose, CA) and corrected for variances.[1,2] Tissue shearing during the staining procedure resulted in regions that could not be registered that were cropped and discarded.

© 2020 Rana A et al. *JAMA Network Open.*

eTable 1. Data Distribution

Patient	Age	Demographics	Tumor	Core (train, test)	Training Patches	Test-G3 Patches	Test-G4 Patches	Test-G5 Patches
1	84	Dominican: Hispanic	G3	6,1	2023	21	-	-
2	59	Unavailable: White	G4, G5 (G4>G5)	2,2	1977	-	-	-
3	61	Unavailable: White	B9, G3, G4 (G3>G4)	3	1068	-	-	-
4	71	European: White	G3	5	1244	-	-	-
5	71	Unavailable: White	G3, G4 (G3>G4)	2	2119	-	-	-
6	71	Unavailable: White	G4	2	1602	-	-	-
7	72	Unavailable: White	G4	3,4	1608	-	292	-
8	72	European: White	B9, G3, G4 (B9>>G3/G4)	3	173	-	-	-
9	59	American: White	B9	1,3	1825	-	-	-
10	73	American: White	G3	4	82	-	-	-
11	60	Unavailable: White	G3	2,2	3522	-	-	-
12	68	Unavailable: White	G3, G4, G5, (G4> G3/G5)	2	1556	-	-	-
13	53	Unavailable: White	G3, G4	2,5	2930	300	744	-
14	51	Unavailable: Black /African American	B9, G4	6	3369	-	-	-
15	72	Unavailable: White	G3, G4	1,3,4	3297	2274	1738	-
16	84	Unavailable: White	G4, G5 (G4>G5)	3,2	5083	-	220	270
17	77	Declined: White	G4	5	4094	-	-	-
18	56	Filipino: White	G3, G4 (G4> G3)	2,6	1161	738	1133	-
19	64	American: White	B9, G3, G4 (G3>G4), Rare G5	2,4	543	505	1351	-
20	63	Unavailable: White	G4, G5 (G4 >G5)	2	716	-	-	-

© 2020 Rana A et al. *JAMA Network Open.*

21	67	Black/African American	G3, G4 (G4 >G3)	6	3549	-	-	-
**Patient**	**Age**	**Demographics**	**Tumor**	**Core (train, test)**	**Training Patches**	**Test-G3 Patches**	**Test-G4 patches**	**Test-G5 patches**
23	52	American: White	G3, G4 (G3>>G4)	5,6	5714	-	-	-
24	79	American: White	G3, G4, G5, (G4> G3/G5)	2	1923	-	-	-
25	62	Unavailable: White	G3, G4 (G3>>G4)	3	1661	-	-	-
26	63	Unavailable: White	G3, G4 (G3>>G4)	2	2725	-	-	-
27	77	Black/African American	G3, G4 (G3>G4)	5,6	3586	-	-	-
28	53	American: White	G4, G5 (G4>G5)	2	1331	-	-	-
29	75	American: White	G3	1,2	905	1351	-	-
30	62	Unavailable: White	G3	2	2025	-	-	-
31	65	White/ Middle Eastern	G3	3	2151	-	-	-
32	67	African: White	G3	1, 2	1005	920	-	-
33	68	Dominican: Other	G4, G5 (G4 > G5)	2	947	-	-	-
34	73	American: White	G4	2,6	1857	-	536	-
35	64	American: White	B9, G3	3	1148	-	-	-
36	76	Declined: White	G4, G3 (G4 >G3)	2	810	-	-	-
37	54	Black / African American	G3	1,2	1151	831	-	-
38	67	Other: White	G3	1	870	-	-	-
**Total**	**-**	**-**	**-**	**-**	**78,322**	**7,019**	**6,146**	**270**

© 2020 Rana A et al. *JAMA Network Open.*

eAppendix 2. Loss Function

Consider I_u and I_s respectively represent the native non-stained and H&E stained image patches in the training dataset. The generator takes in I_u as the input and generates I_{cs}, the corresponding computationally stained image patch, as the output. The discriminator analyses the output image, I_{cs}, and predicts the probability that I_{cs}, is real (from the training dataset) or fake (output from generator). The loss function consisted of the cGAN loss,[3] a L1 component and a PCC factor between I_s and I_{cs}. The loss equation was:

$$\mathcal{L}_{cGAN}(G,D) = E_{x,y}[logD(x,y)] + \alpha E_{x,y}[\log{(1 - D(x,G(x,z)))}]$$

$$\mathcal{L}_{cGAN}(G,D) = E_{x,y,z}[\|y - G(x,z)\|_1]$$

$$\mathcal{L}_{PCC}(G) = E_{x,y,z}[PCC(y,G(x,z))]$$

The final loss function is:

$$G^* = arg \min_G \max_D \mathcal{L}_{cGAN}(G,D) + \lambda \mathcal{L}_{L1}(G) + \gamma \mathcal{L}_{PCC}(G)$$

Where *x* is the input image, *y* is the target image and *z* is the random noise, added as dropout in our work. $\mathcal{L}_{cGAN}(G,D)$ is the $cGAN$ loss function, $\mathcal{L}_{L1}(G)$ is the $L1$ loss between the output of the generator and the target image, and $\mathcal{L}_{PCC}(G)$is the proposed term that calculated the Pearson's correlation coefficient between the generator output and target image. α=1, λ=100 and γ =10 gave best results. After training, the model accepted unseen native non-stained image patches and generated computationally H&E stained images patches.

Technical implementation of the CGAN model: The discriminator was trained after every single training step for the generator. Both networks were trained for 10 epochs each using Adam optimization[12], and a batch size of one on a NVIDIA GeForce 1080 TI GPU (NVIDIA, Santa Clara, CA) with 12 GB of VRAM and CUDA acceleration to speed up training. One epoch of training took approximately 16 GPU hours. The patches were randomly flipped and dropped out to prevent overfitting and increase generalization capability of the model.

© 2020 Rana A et al. *JAMA Network Open.*

eAppendix 3. Interpretation

Methods: Non-overlapping patches were cropped from these four sets of images, resulting in four sets of around 2000 patches (of size 1024x1024 pixels each). Patches not containing any tissue were discarded, resulting in 448 patches for each set. These four sets of patches were fed into trained staining and destaining models. Nonstained and destained patches were fed into computational staining model, while computationally and H&E stained patches were fed into the computational destaining model. The activation maps generated after each layer block (lrelu – conv2d - batch norm) were saved for further analysis. Activation maps for selected patches were analyzed to understand the transformation of input images as they passed through the generator neural network layer, and to identify which convolutional kernels are activated. Activation maps generated by the top five most activated kernels were extracted, ranked and visualized by heatmaps. Activation maps for each layer were ordered in descending order (ranked by numbers of pixels of intensity greater than 200). Top five activation maps (heatmap) from each layer are shown in Figure 3 (benign input patch) and eFigure 3 (Gleason grade 3 tumor patch). Heatmaps were the standard 'jet' heatmaps plotted using matplotlib library in python.

The activation maps, for an input image, for each layer were rescaled to 0-1 and resized to a standard size (128x128 pixels) and concatenated into a grid (16 by K grid, where k is variable and is equal to number of patches divided by 16). This process was done for every one of the 448 patches for all four image sets. The activation maps for the matching patches (unstained-destained and computationally stained-H&E stained input patch pairs) were compared and the mean squared error (MSE) was calculated. The MSE plot for the two sets of matching input patches can be seen in eFigure 5.

Results: The top five activation maps (heatmaps) obtained from the first five layers (rows I-V) and the last four layers (rows 16-19) of the generator (excluding the input and output layers) are presented in Figure 3 and eFigure3 and 4. The first five rows represent the first 5 layers of the generator and the last 4 rows represent the last four layers of the generator. It is evident from the activation heatmaps that the network does a good job of extracting the tissue from the background (eFigure 3, and 4: L1-I and L1-III). eFigure 3 and 4: L1-II, L1-III and L2-II, L2-IV and L2-V indicated that the model learnt to recognize some features in the tissue. As the image passed through the layers, we can see localized high activations (bright red and orange color), indicating region of interest learnt by the trained generator. While the initial layers learn low level features like tissue, background, circles and simple patterns, deeper layers learn to recognize high-level patterns (using a combination of the knowledge accumulated by the previous layers). It is difficult to understand these visualizations and they look like noise to the human eye. Layers 16 through 19 show the activation heatmap from the decoder side of the generator (Figure 3 and efigures 3 and 4). These layers try to decode the encoded information back to the original size (1024x1024 output), while in the process computationally staining the image."

In eFigure 5 the blue lines represent the flow of MSE value through the layers for all 448 patch pairs unstained-destained (left) and H&E stained - computationally stained (right). The green and orange lines are the first and the third quartile values. We can see the variance between the MSE values at all the layers for the 448 patches. The variance is higher for the encoder layers and is less for the decoder layers. This the case for both unstained-destained plot and H&E stained - computationally stained plot. Peaks were observed at layers 3, 10 and 17.

© 2020 Rana A et al. *JAMA Network Open.*

eFigure 1. Color Coded Overlaid Validation Images

In Figures 1- 13, each figure contains (a) Ground truth Hematoxylin and Eosin (H&E) dye stained RGB Whole Slide Images (RWSI); (b) Computationally H&E stained RWSI; (c) Computationally H&E stained RWSI overlaid with colors representing comparisons between true positive (green), false positive (red) and false negative (blue) of IOU of healthy and tumor annotations provided by five physicians on ground truth H&E dye and computationally H&E stained prostate core biopsy images; (d) Ground truth native non-stained RWSI image; (e) Computationally destained RWSI.

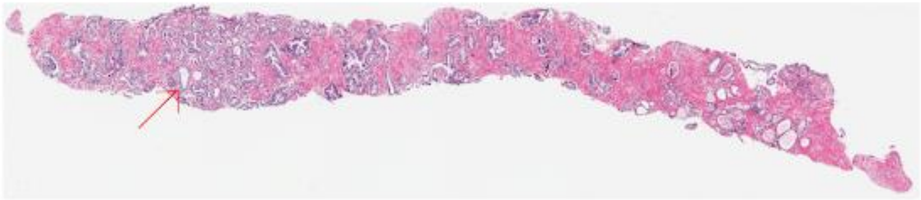

(a)

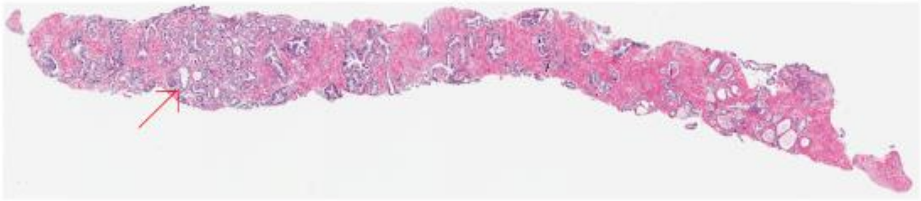

(b)

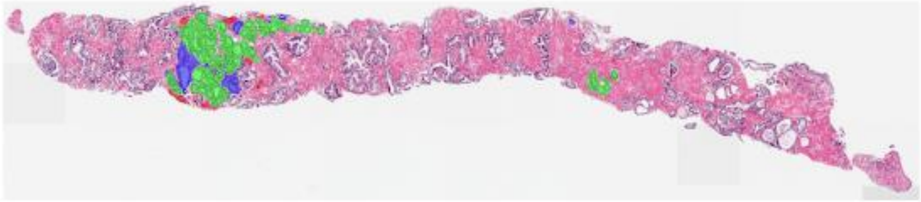

(c)

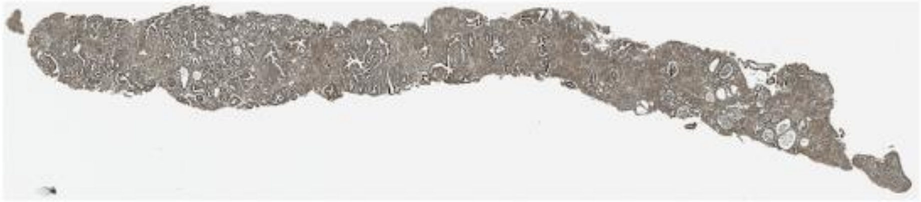

(d)

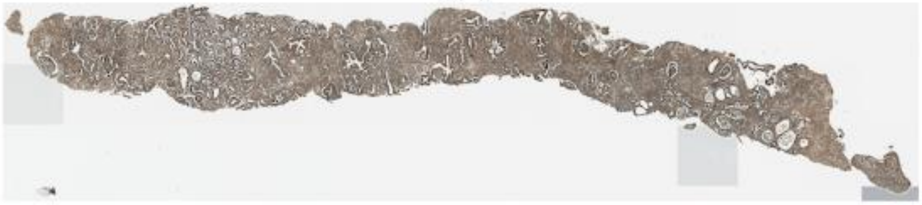

(e)

eFigure 1.1 (a) Ground truth H&E dye stained RWSI with arrow highlighting atypical gland indeterminate for malignancy; (b) Computationally H&E stained RWSI with arrow showing atypical gland with preserved morphology as shown on image in panel (a).

© 2020 Rana A et al. *JAMA Network Open.*

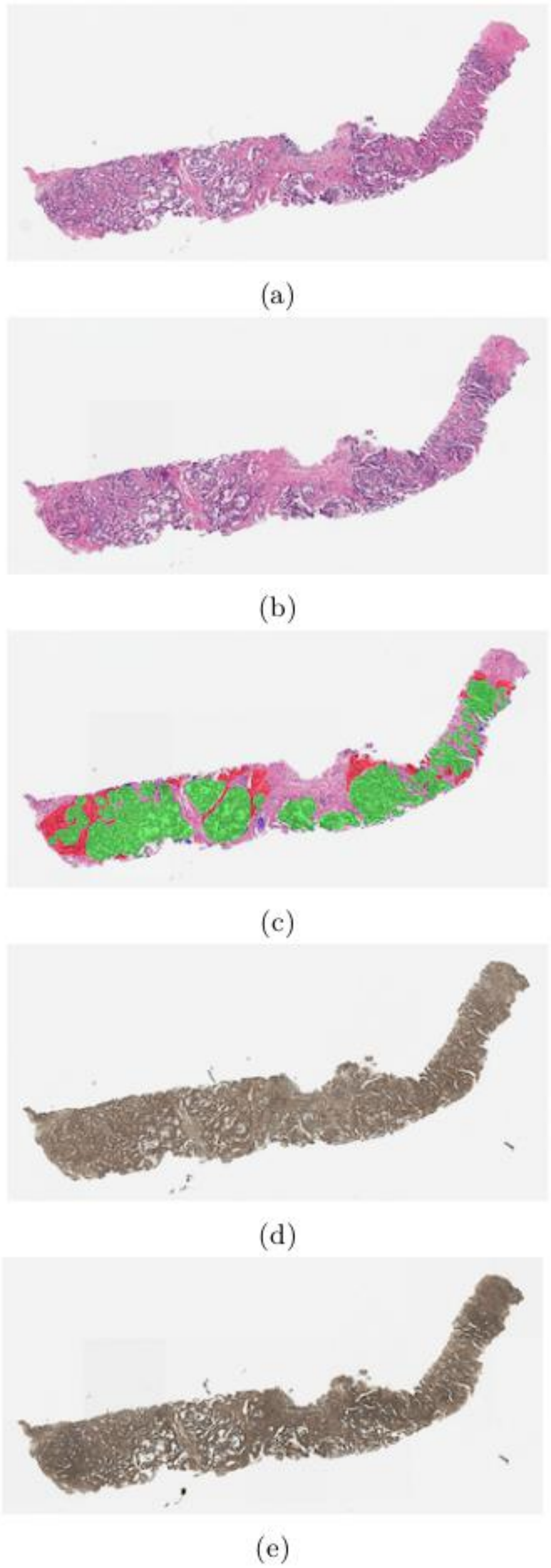

eFigure 1.2

© 2020 Rana A et al. *JAMA Network Open.*

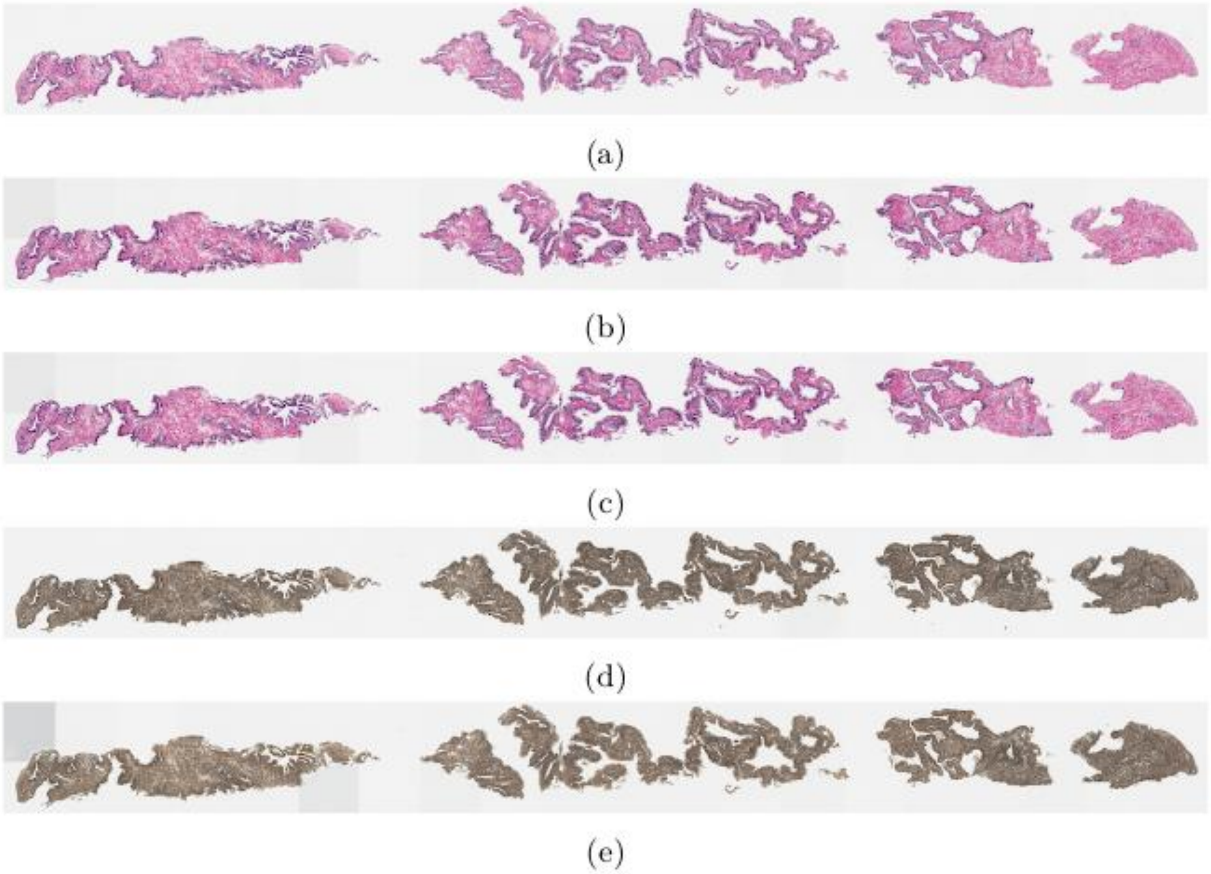

eFigure 1.3

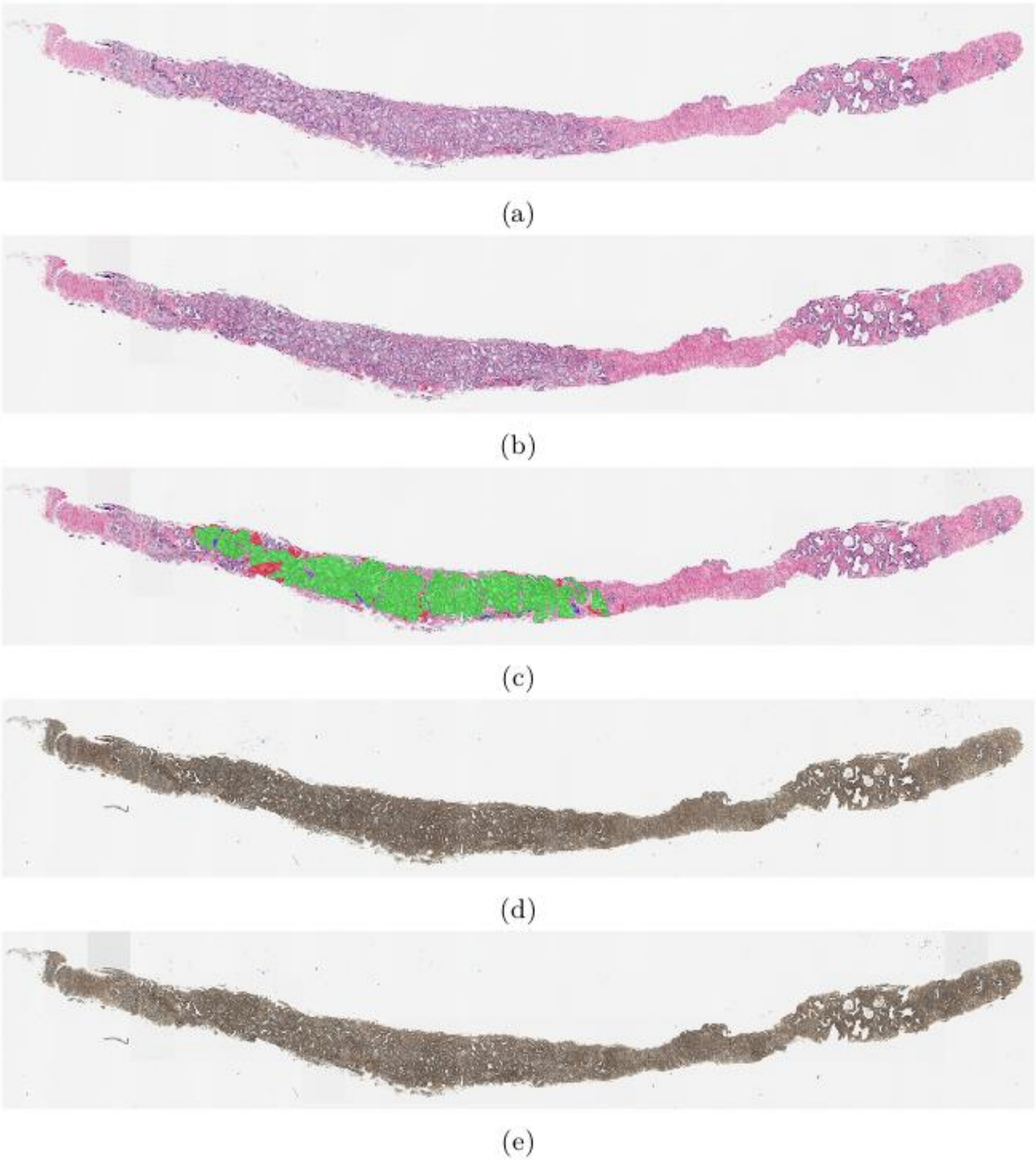

(a)

(b)

(c)

(d)

(e)

eFigure 1.4

© 2020 Rana A et al. *JAMA Network Open.*

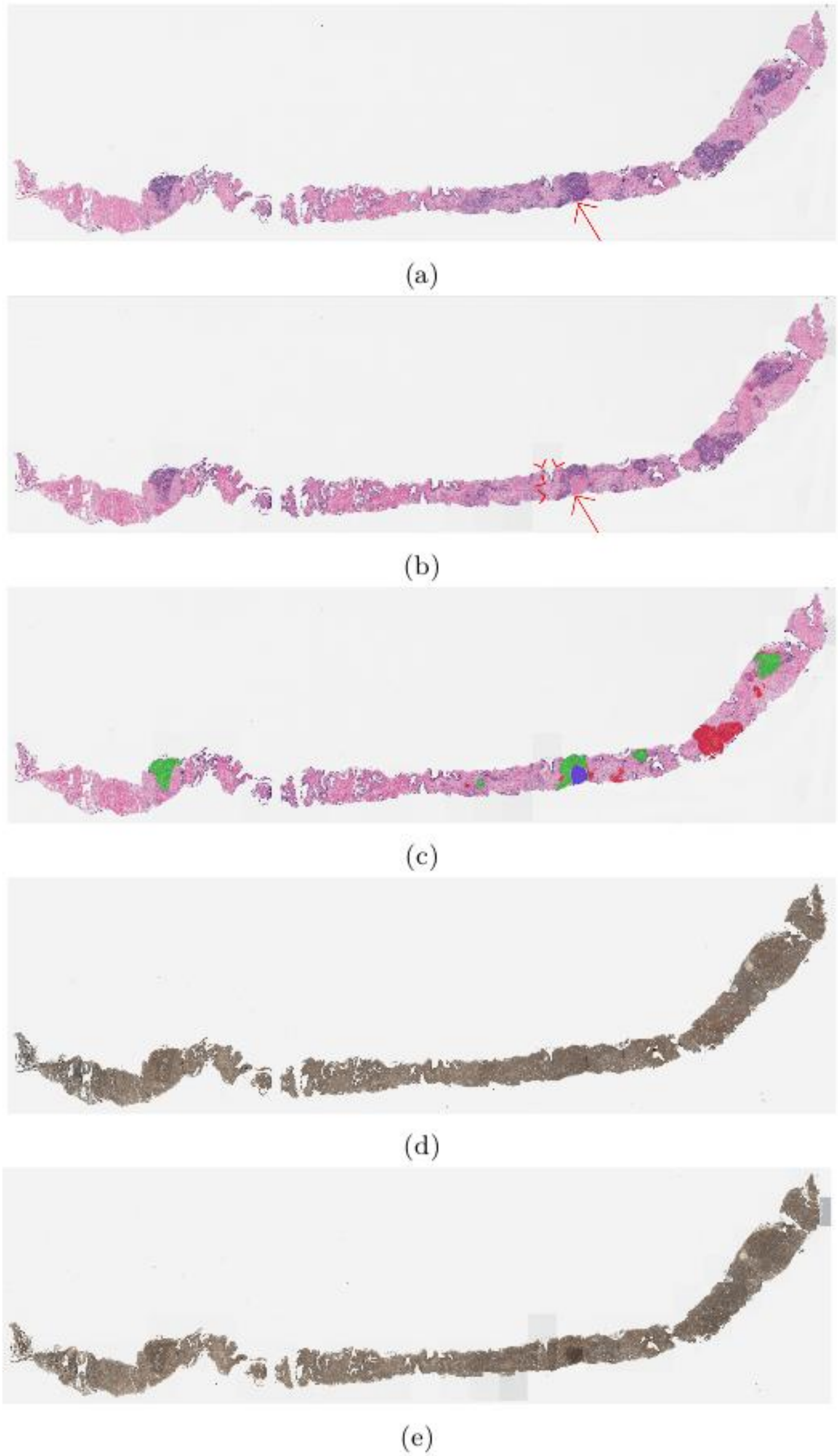

eFigure 1.5 (a) Ground truth H&E dye stained RWSI showing comedo necrosis (arrow); (b) Computationally H&E stained RWSI where malignant glands are well preserved (arrowheads), comedo necrosis is not evident (arrow).

© 2020 Rana A et al. *JAMA Network Open.*

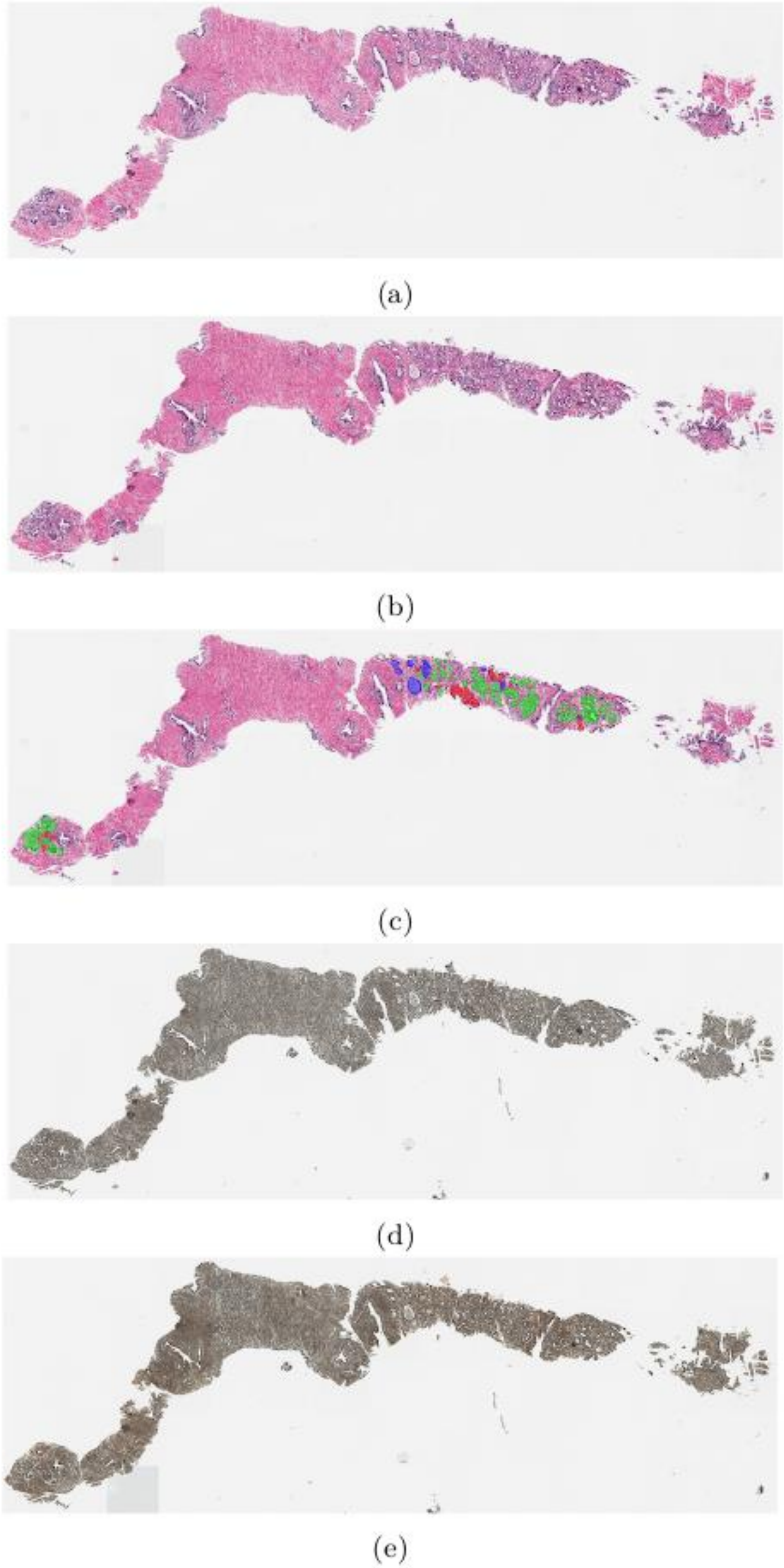

eFigure 1.6

© 2020 Rana A et al. *JAMA Network Open.*

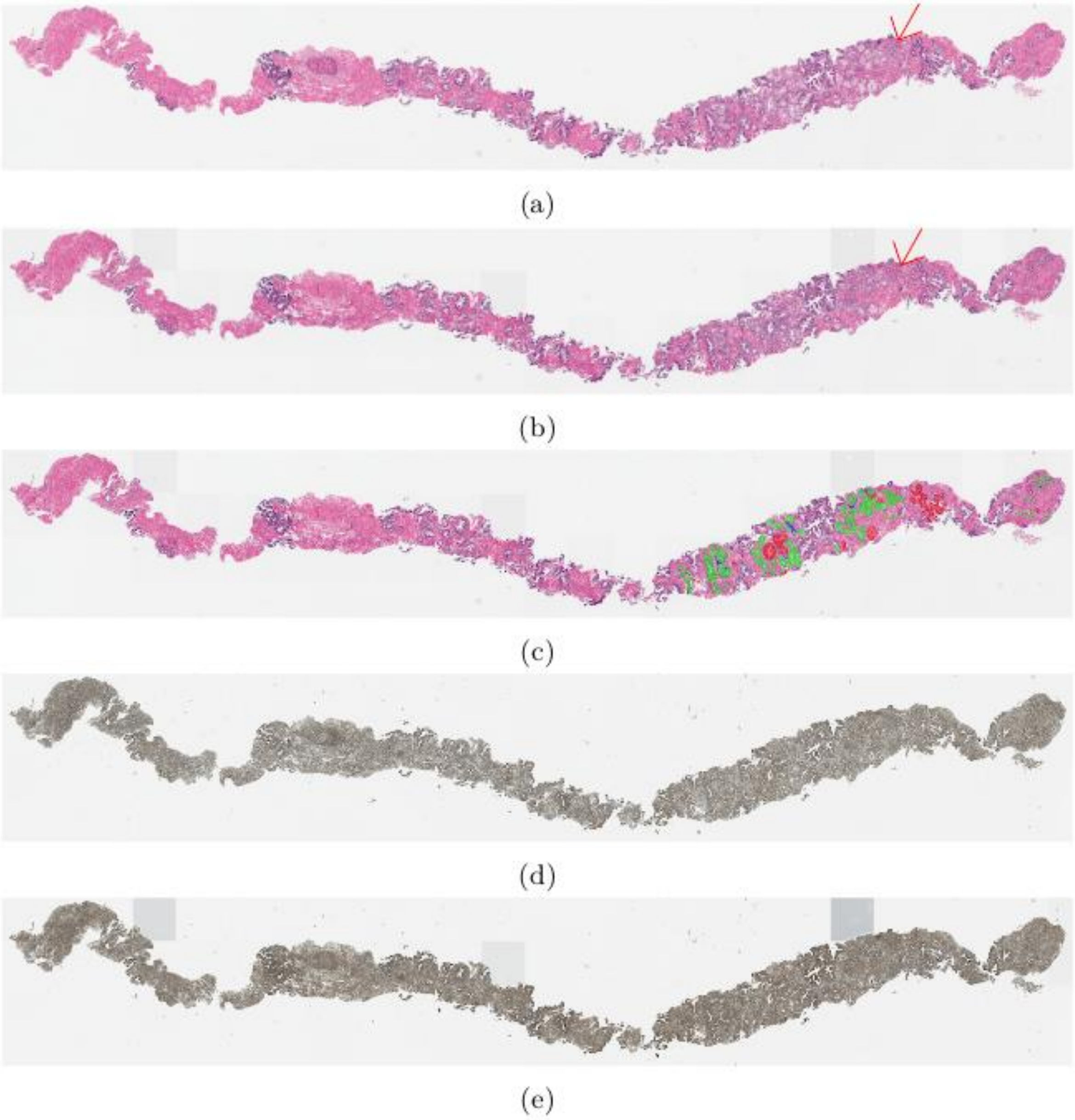

eFigure 1.7 (a) Ground truth H&E dye stained RWSI with arrow showing tumor; (b) Computationally H&E stained RWSI with arrow showing tumor.

© 2020 Rana A et al. *JAMA Network Open.*

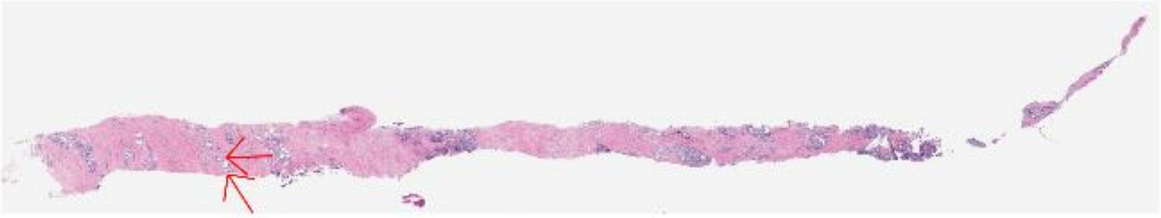

(a)

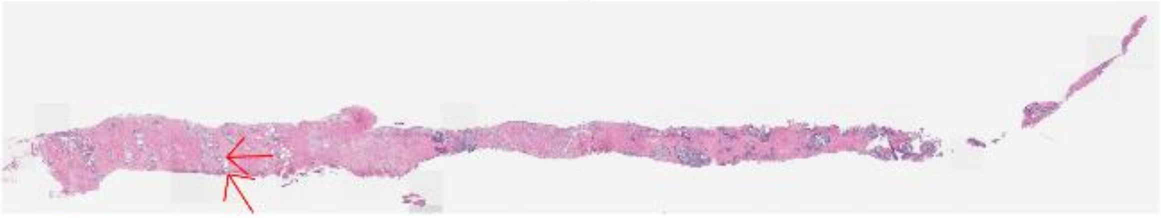

(b)

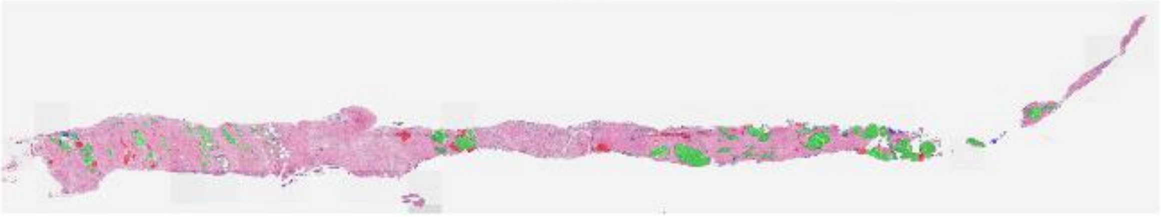

(c)

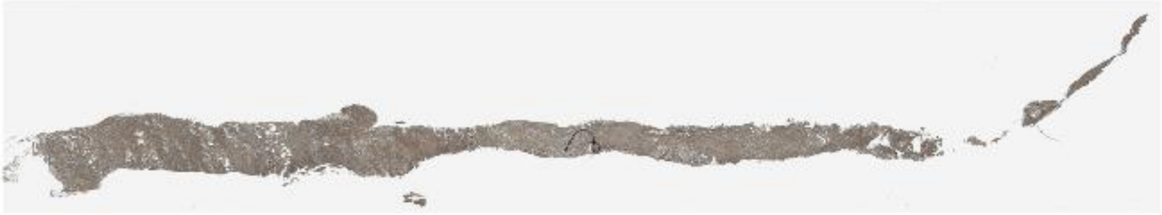

(d)

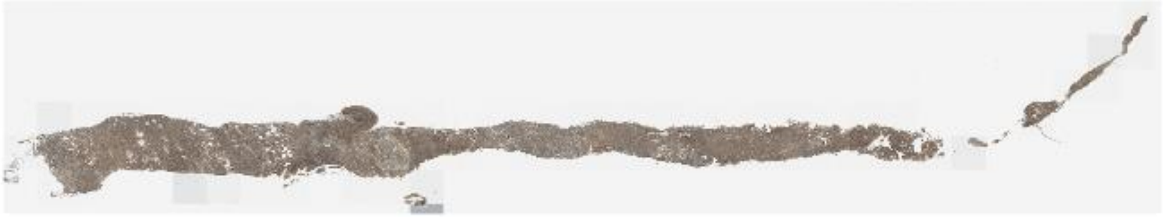

(e)

eFigure 1.8 (a) Ground truth H&E dye stained RWSI with arrows indicating rare poorly formed glands; (b) Computationally H&E stained RWSI with arrows indicating rare poorly formed glands.

© 2020 Rana A et al. *JAMA Network Open.*

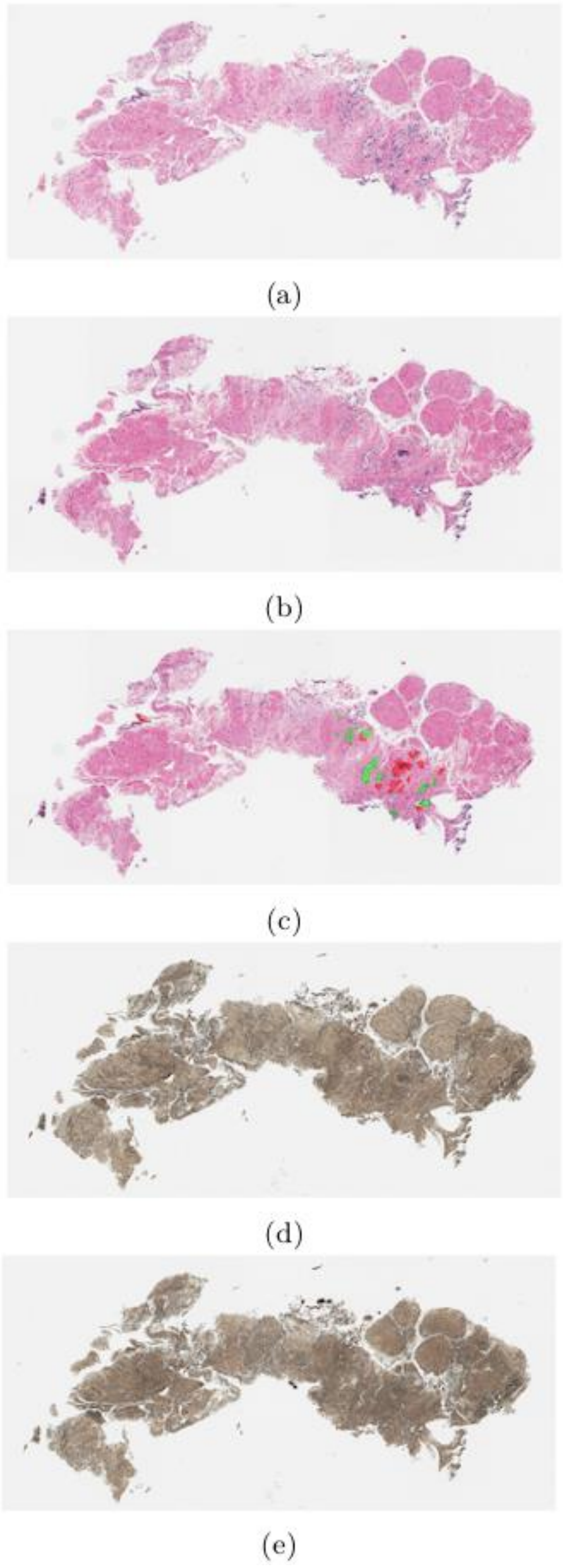

(a)

(b)

(c)

(d)

(e)

eFigure 1.9

© 2020 Rana A et al. *JAMA Network Open.*

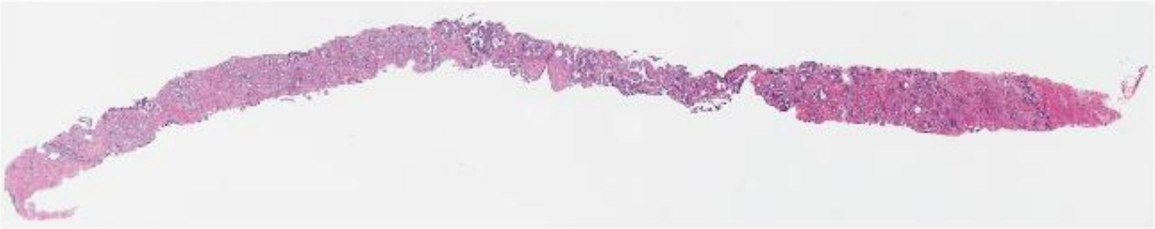

(a)

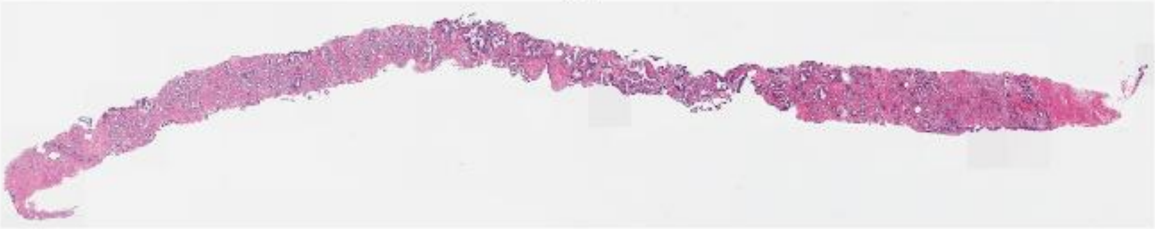

(b)

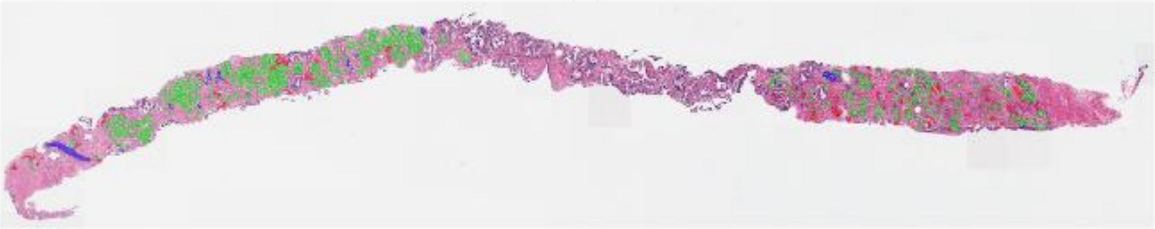

(c)

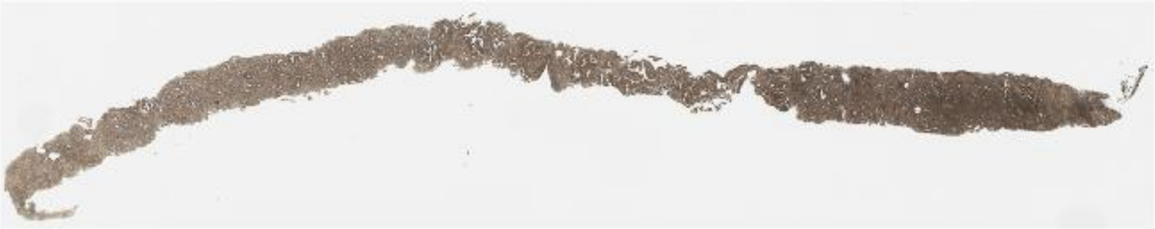

(d)

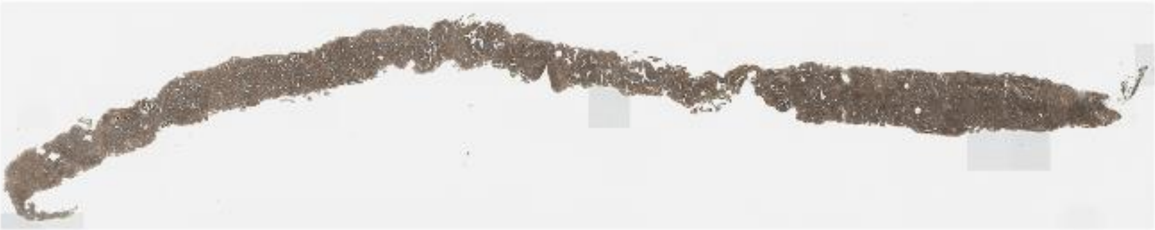

(e)

eFigure 1.10

© 2020 Rana A et al. *JAMA Network Open.*

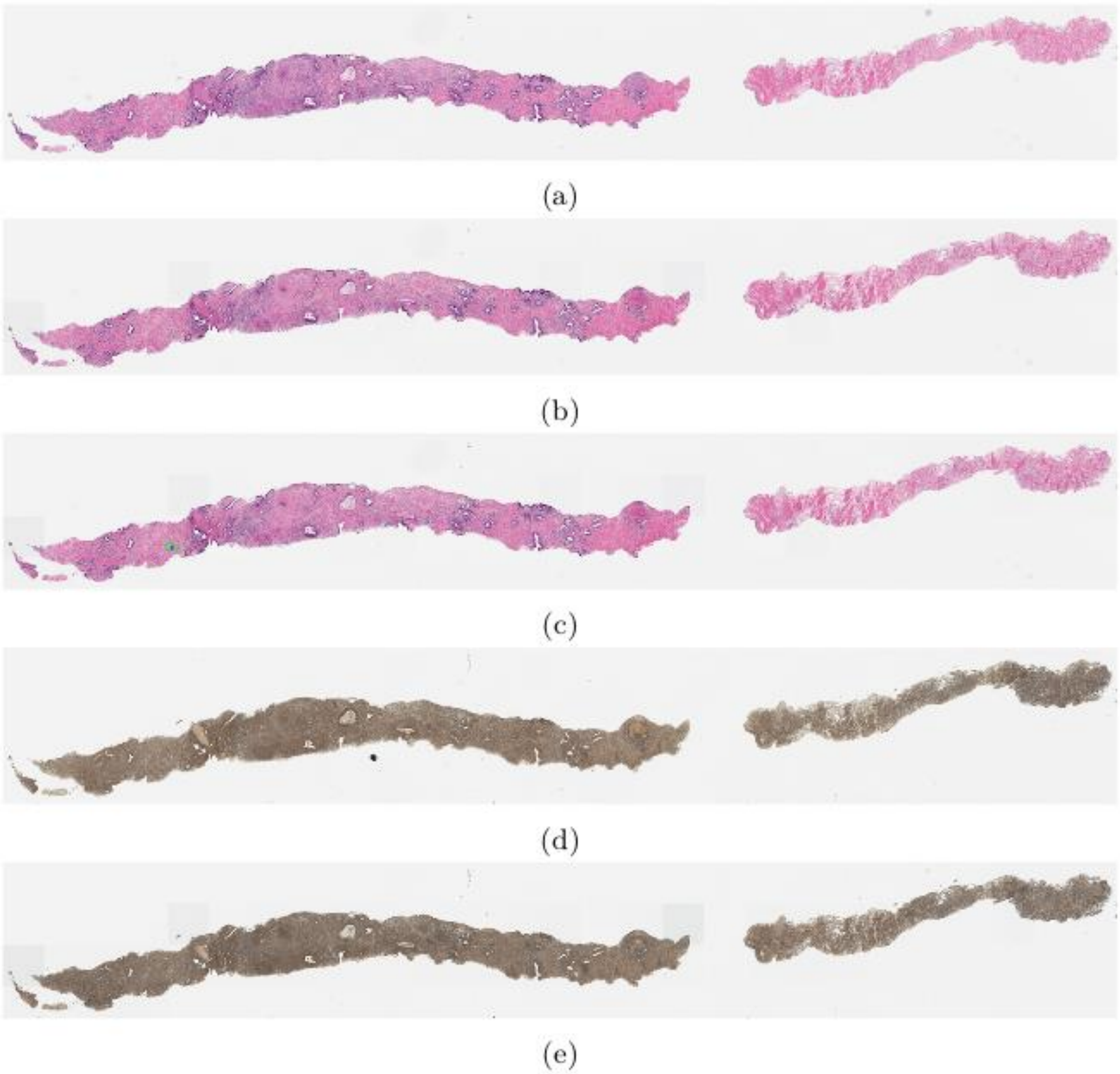

eFigure 1.11

© 2020 Rana A et al. *JAMA Network Open.*

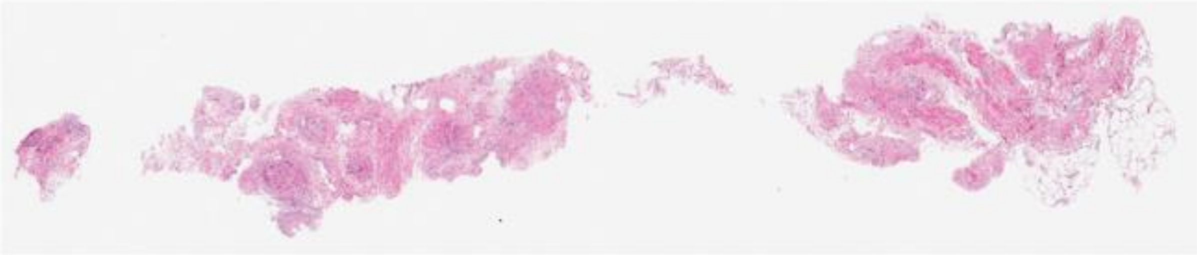
(a)

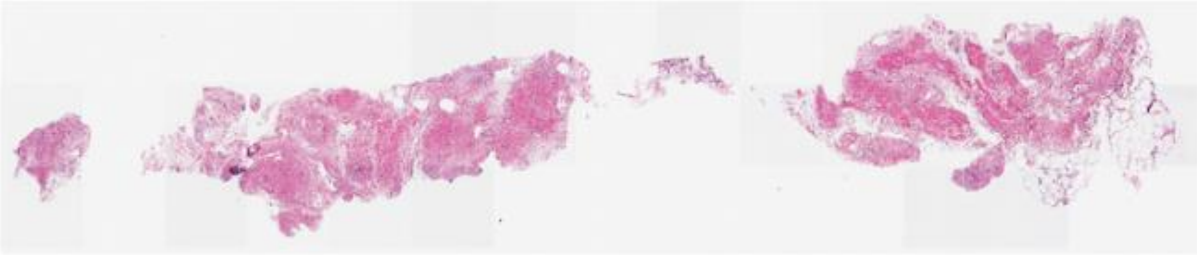
(b)

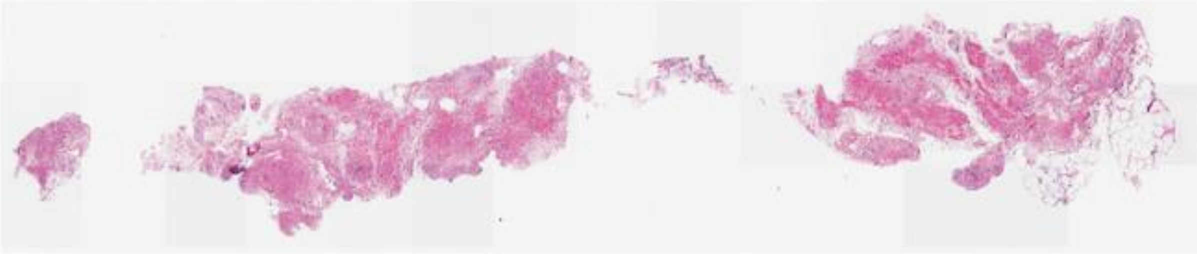
(c)

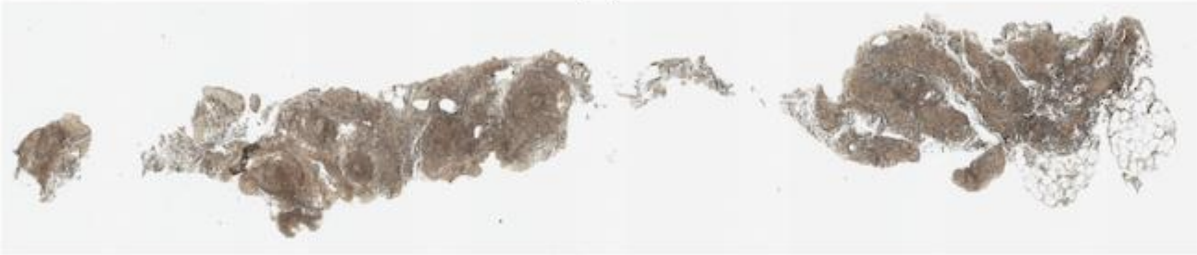
(d)

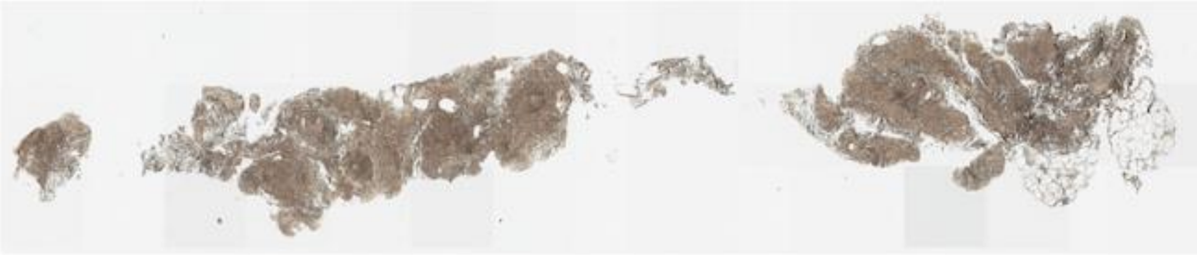
(e)

eFigure 1.12

© 2020 Rana A et al. *JAMA Network Open.*

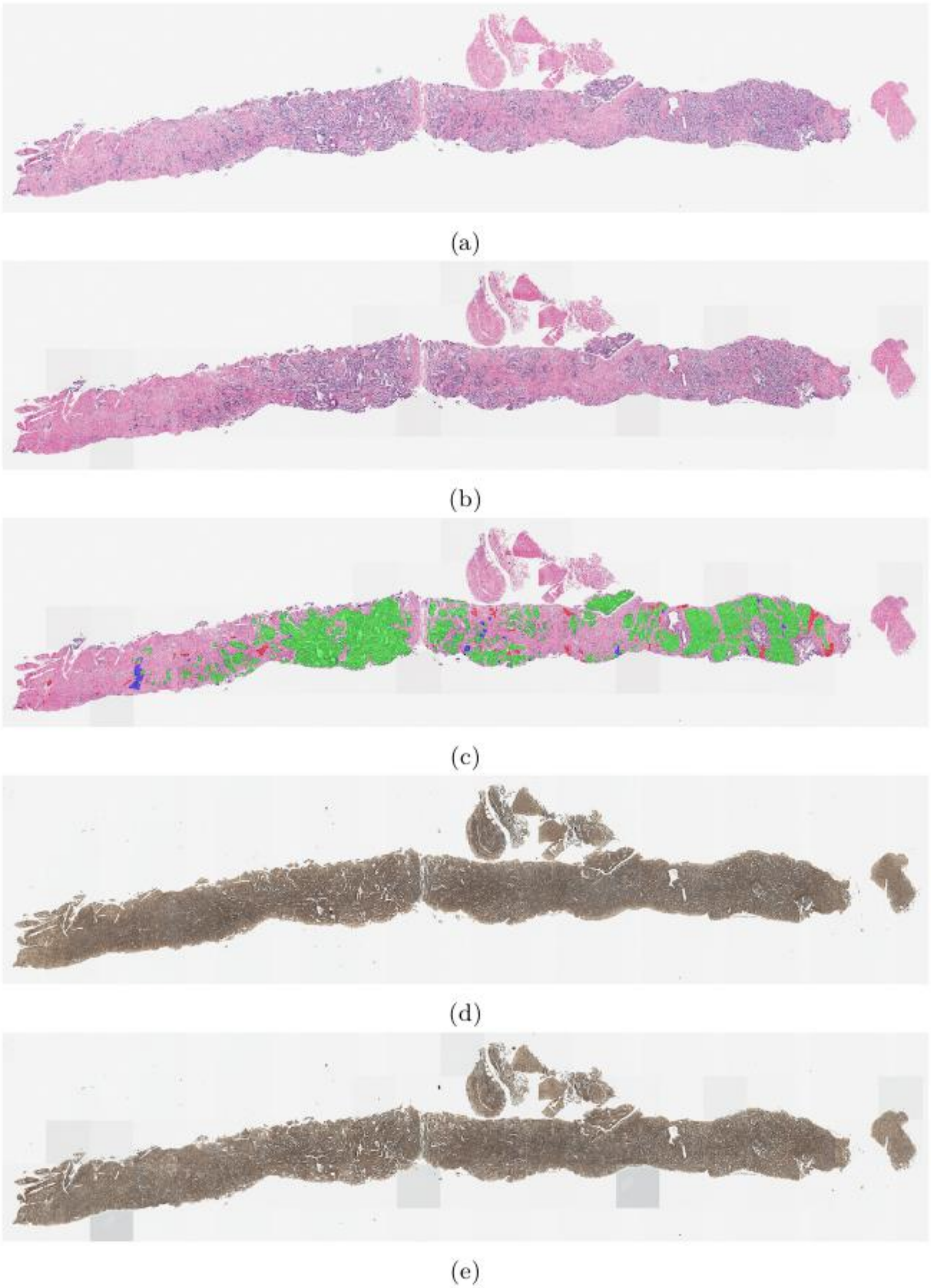

eFigure 1.13

© 2020 Rana A et al. *JAMA Network Open.*

eFigure 2. Visualization and Explanation of Computational Hematoxylin and Eosin Staining Process by Custom Autoencoder Neural Network

Panel (a) Processing of native non-stained prostate core biopsy images as various layers of the encoder and decoder neural networks computationally stain them. The blue boxes represent hidden activation layers of the neural network. Panel (b) A single input native non-stained patch and representative concatenated activation maps from the corresponding hidden layers in panel (a) of kernels of the decoder neural network as it flows through them, are shown.

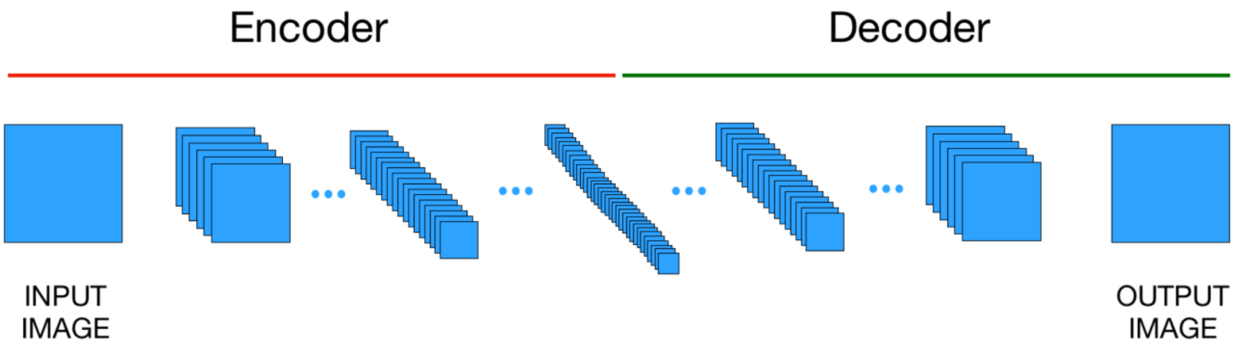

(a)

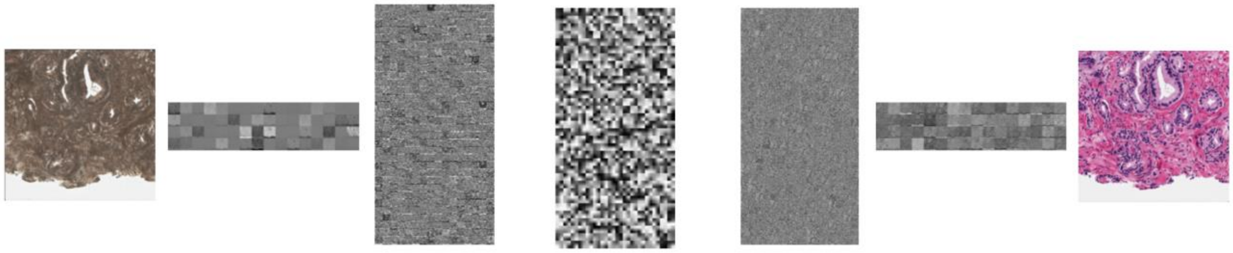

(b)

© 2020 Rana A et al. *JAMA Network Open.*

eFigure 3. Activation Maps of Kernels of Trained Generator Neural Network Model Layers

Activation maps of kernels of trained generator neural network model layers after feeding a native non-stained prostate core biopsy image patch with Gleason grade 3 tumor as it gets computationally Hematoxylin and Eosin stained. Rows show top five activation maps from layers L1 - L5 and L16 - L19 arranged in decreasing order of their activations from left to right (columns I-V).

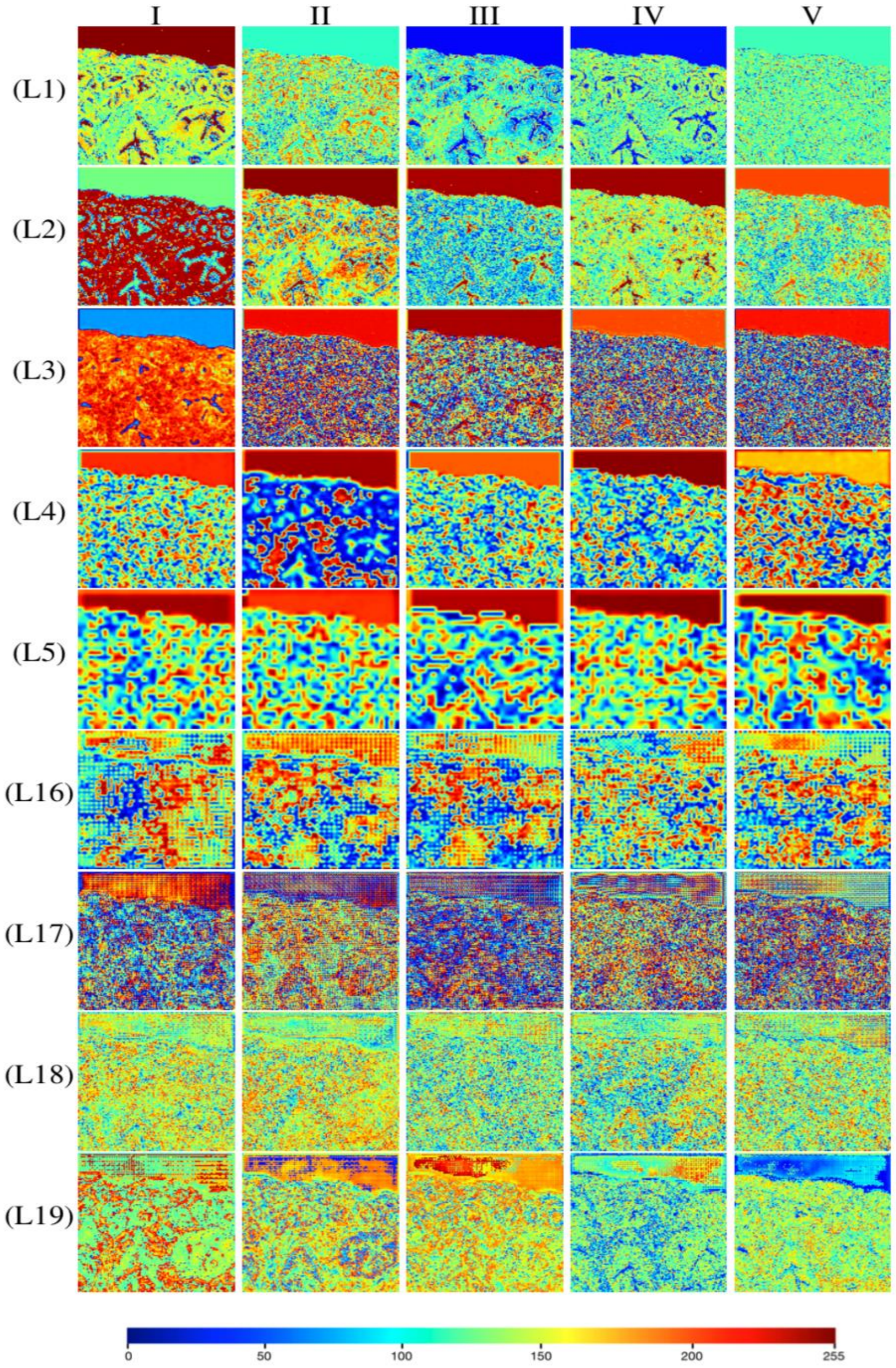

© 2020 Rana A et al. *JAMA Network Open.*

eFigure 4. Activation Maps of Kernels of Various Generator Neural Network Layers After Entering Hematoxylin And Eosin Dye–Stained Patch With Gleason Grades 4 And 5 Prostate Tumor

Panel (a) and (b) show Gleason grade 4 and 5 respectively. Rows show top five activation maps from layers L1 - L5 and L16 - L19 arranged in decreasing order of their activation from left to right (columns I-V).

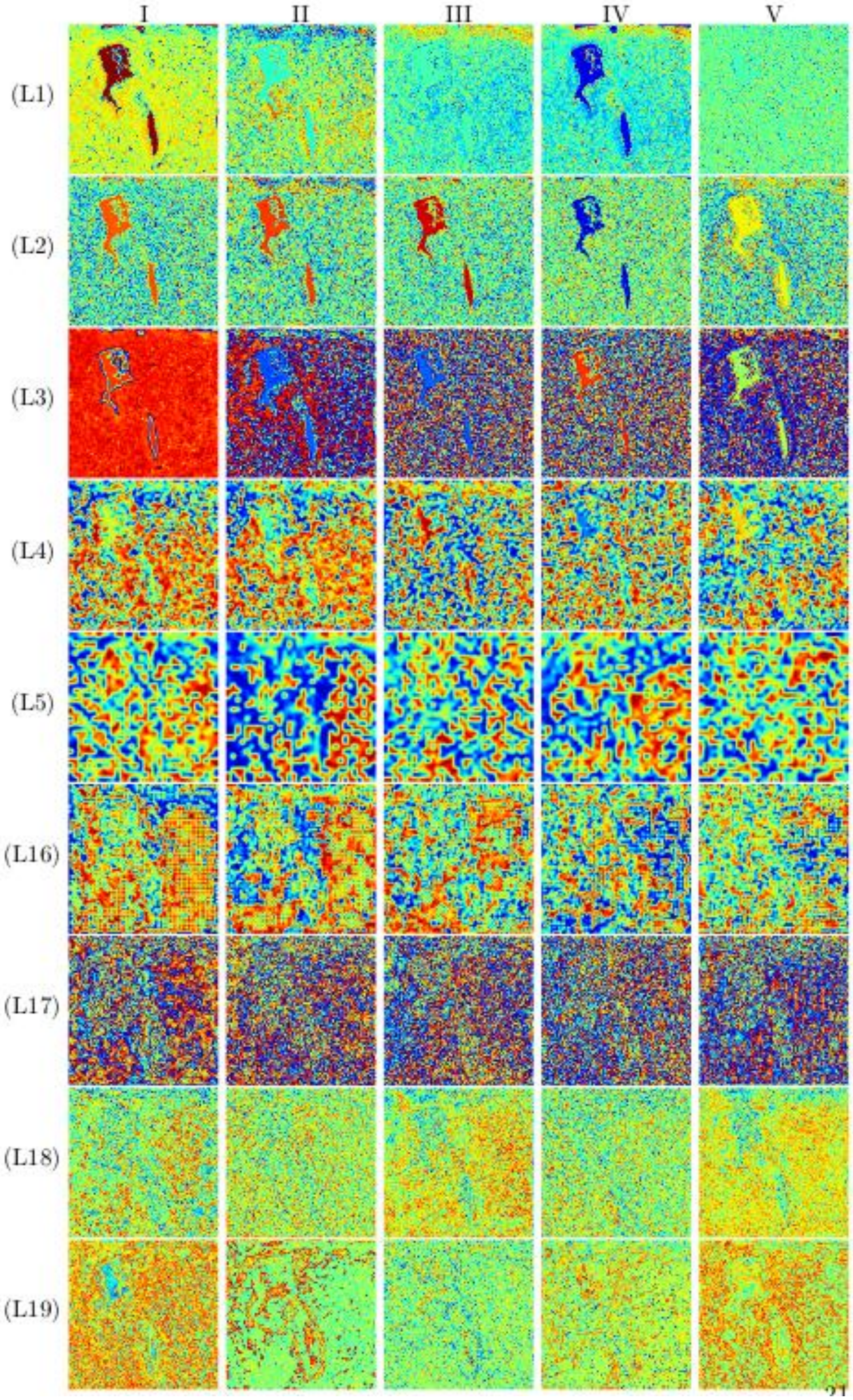

© 2020 Rana A et al. *JAMA Network Open.*

(a)

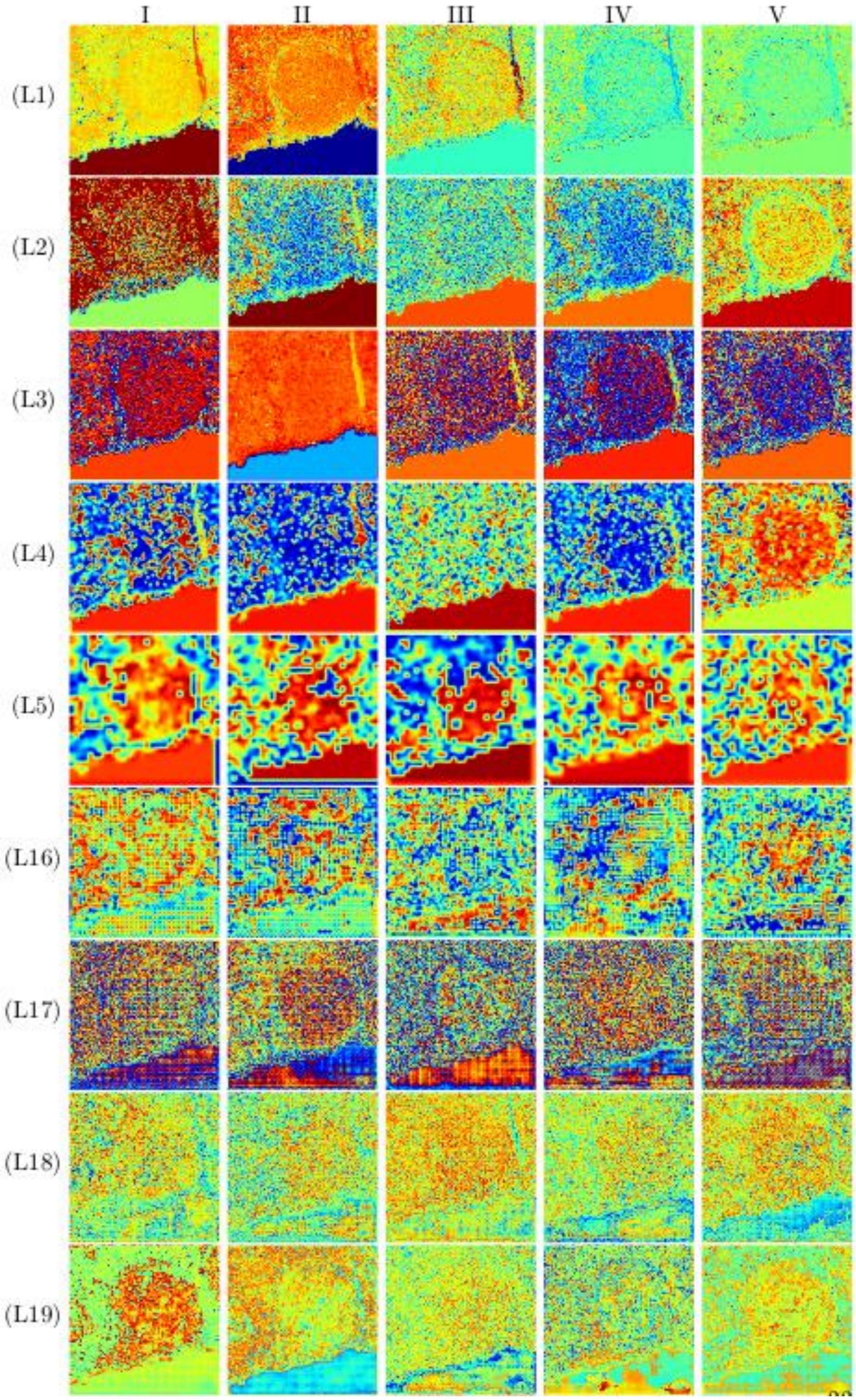

(b)

© 2020 Rana A et al. *JAMA Network Open.*

eFigure 5. Comparison of Mean Squared Errors Between Kernel Activation Maps of Pairs of 448 Validation Image Patches Generated by the Trained Neural Network Models

(a) MSE of ground truth native non-stained and corresponding computationally destained input patch activation maps generated by the trained computational staining model; (b) MSE of computationally Hematoxylin and Eosin (H&E) stained – and corresponding ground truth H&E dye stained matching input patch activation maps generated by the trained computational destaining model. Blue lines represent the MSE values for each of the 448 input pairs. Red curve represents average MSE value at each layer of the generator for all input pairs. The green and orange curves represent the first and third quartile MSE values for all input patch pairs. Lower MSE indicates more accuracy between the activation maps being compared.

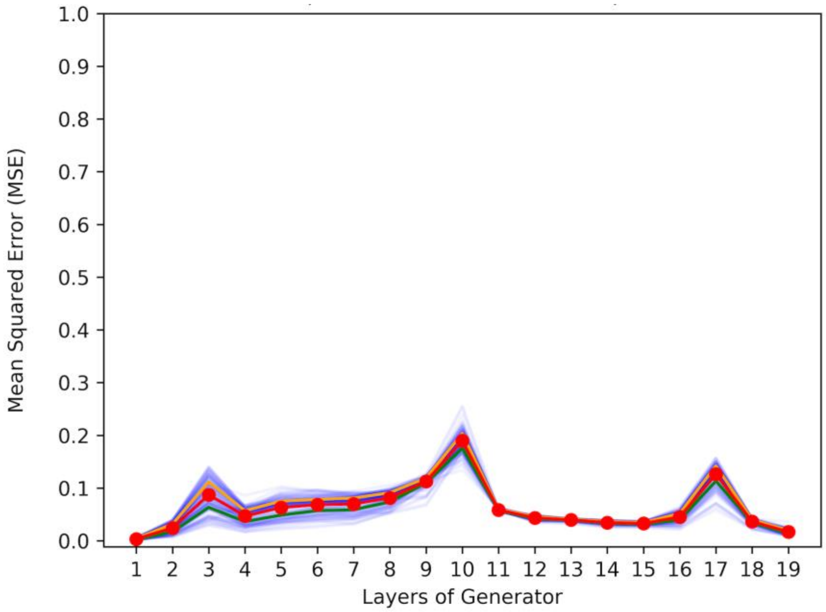

(a)

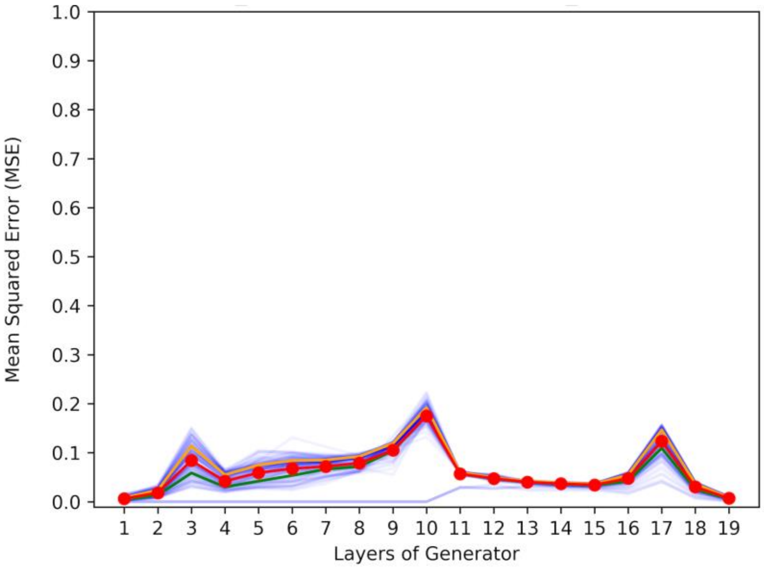

(b)

© 2020 Rana A et al. *JAMA Network Open.*

eTable 2. Mean Pixel Intensity Following Computational Staining and Destaining

Average pixel intensity differences following computational staining and destaining: Difference between Native non-stained and computationally stained (U_C); native non-stained and H&E dye stained (U_H); ground truth H&E dye stained and computationally stained (H*_C); H&E dye stained and computationally destained (H_D); H&E dye stained and native non-stained (H_U); computationally destained and ground truth native non-stained (D_U*). All values are in pixel intensities (0 to 255) calculated by subtracting the 2nd from 1st image. Positive values indicate decrease in average pixel intensities and negative values indicate gain. Values have been rounded to nearest integer. H is H&E dye stained image, C is computationally stained image, D is computationally destained image and U is native non-stained image. Ground truth images are indicated with '*' to facilitate comparisons with computational images when necessary.

**Image**	**Computational staining**			**Computational destaining**		
	**U_C (±std)**	**U_H(±std)**	**H*_C(±std)**	**H_D(±std)**	**H_U(±std)**	**D_U*(±std)**
1	-42	-43	1	45	43	-3
2	-30	-26	-4	37	26	-11
3	-39	-47	8	40	47	1
4	-48	-49	1	48	49	1
5	-48	-44	-3	44	44	0
6	-32	-34	2	44	34	-10
7	-19	-21	1	32	21	-12
8	-53	-53	0	58	53	-5
9	-45	-48	3	56	48	-8
10	-42	-40	-2	44	40	-4
11	-43	-42	-2	48	42	-6
12	-56	-66	10	62	66	4
13	-50	-58	8	56	58	2
**MEAN**	**-42±10**	**-44±12**	**2±4**	**47±8**	**44±12**	**-3±5**

© 2020 Rana A et al. *JAMA Network Open.*

eTable 3. Change in Mean Pixel Intensity in Red, Green, and Blue Channels per Image

ST1: Change in average pixel intensity (Red channels) per image: Native non-stained to computationally stained image (U_C), Native non-stained to Hematoxylin and Eosin (H&E) dye stained image (U_H), H&E dye stained to computationally restained (H_C). All values are in pixel intensities (0 to 255) calculated by subtracting the 2nd image from 1st image. Positive values indicate drop in average pixel intensity and negative values indicate gain in pixel intensity. H is H&E dye stained image; C is computationally stained images and U is native non-stained image. Values have been rounded to nearest integer.

Image	U_C(± std)	U_H (±std)	H_C(±std)
1	-66	-66	0
2	-38	-36	-3
3	-51	-59	7
4	-58	-57	0
5	-60	-53	-7
6	-60	-59	-1
7	-48	-50	2
8	-68	-67	-1
9	-62	-61	-1
10	-55	-49	-6
11	-57	-54	-3
12	-75	-81	6
13	-59	-62	3
**MEAN**	**-58±9**	**-58±10**	**0±4**

ST2: Change in average pixel intensity (Green channels) per image: Native non-stained to computationally stained image (U_C), Native non-stained to Hematoxylin and Eosin (H&E) dye stained image (U_H), H&E dye stained to computationally restained (H_C). All values are in pixel intensities (0 to 255) calculated by subtracting the 2nd image from 1st image. Positive values indicate drop in average pixel intensity and negative values indicate gain in pixel intensity. H is H&E dye stained image; C is computationally stained images and U is native non-stained image. Values have been rounded to the nearest integer.

Image	U_C (±std)	U_H (±std)	H_C (±std)
1	-2	-2	0
2	6	11	-6
3	-2	-12	10
4	-15	-16	1
5	-14	-11	-3
6	10	6	4
7	24	24	0
8	-23	-22	-1
9	-11	-15	4
10	-5	-4	-1
11	-6	-4	-3
12	-23	-35	12
13	-19	-29	11
**MEAN**	**-6±13**	**-8±16**	**2±5**

© 2020 Rana A et al. *JAMA Network Open.*

ST3: Change in average pixel intensity (Blue channels) per image: Native non-stained to computationally stained image (U_C), Native non-stained to Hematoxylin and Eosin (H&E) dye stained image (U_H), H&E dye stained to computationally restained (H_C). All values are in pixel intensities (0 to 255) calculated by subtracting the 2nd image from 1st image. Positive values indicate drop in average pixel intensity and negative values indicate gain in pixel intensity. H is H&E dye stained image; C is computationally stained images and U is native non-stained image. Values have been rounded to nearest integer.

Image	U_C (±std)	U_H (±std)	H_C (±std)
1	-58	-60	2
2	-58	-55	-4
3	-62	-70	7
4	-71	-74	3
5	-70	-69	-1
6	-46	-49	3
7	-34	-35	2
8	-70	-71	1
9	-64	-68	4
10	-66	-67	1
11	-67	-68	1
12	-72	-82	11
13	-73	-83	10
**MEAN**	**-62±11**	**-65±13**	**3±4**

© 2020 Rana A et al. *JAMA Network Open.*

eTable 4. Intrarater Agreement Calculated on Dye Stained and Computationally Stained Images

Intra-rater agreement calculated on Hematoxylin and Eosin (H&E) dye stained RGB Whole Slide Images (RWSI) and computationally stained RWSI using Intersection over Union (IoU). `-' indicates that the tumor (or tumor grade) label was not provided by the pathologists. Higher IoU score is better with a score of 1.0 representing perfect match of labels.

Image	H&E dye RWSI (±std)	Computationally stained RWSI (±std)
1	0.83	0.59
2	0.85	0.75
3	-	-
4	0.73	0.75
5	0.84	0.70
6	0.78	0.89
7	0.76	0.78
8	0.71	0.73
9	0.79	0.80
10	0.76	0.78
11	0.97	0.87
12	-	-
13	0.82	0.83
**MEAN**	**0.81±0.07**	**0.77±0.08**

Statistical analysis using t-test (p value~0.28>0.005) for the intra-IOU values shows no significant difference between the mean of intra-IOU of H&E stained (mean~0.81, std~0.072) and Computationally stained images (mean~0.77, std~0.08).

© 2020 Rana A et al. *JAMA Network Open.*

eAppendix 4. Evaluation of the Activation Maps of Trained Deep Neural Network

Clinical evaluations of computationally stained images: Figure 2 in the manuscript shows representative input non-stained image patches in row (a) that had Gleason grade 3 (columns I, II) or 4 (columns III, IV) tumors or were benign (column V), and their computational H&E staining (row c) and accuracy calculated using annotations by multiple physicians (row d). Tissue morphology in computationally stained patches (row c) matches closely with H&E dye stained patches (row b). Patch c-I successfully generated a benign area along with tumor signature (as indicated by arrows) and confirmed in row d-I. Computationally stained patches (row c) retain appearance of benign and malignant glands and stroma seen in H&E dye stained patches (row b). Patch b-III also contains edge/crush artifact (arrowheads) that is preserved in computationally stained image (row c-III). Same patches are shown (row d) with color-coded areas of agreement and disagreement between the labels provided on H&E dye stained images and computationally stained RWSI. It is evident that the computationally H&E stained patches represent tumor signatures with high accuracy and pathologists are able to correctly identify tumor. Majority of observed disagreements between raters did not represent misidentification of glands as benign or malignant. Instead, they show differences in rater annotation at borders of tumor labels, mainly due to differences in labeling style with some raters providing course labels and others annotating detailed labels (row d-III, arrows), or biopsy edges, as some raters chose to score partial/crushed glands at the periphery of samples and others did not (row d-III, arrowheads). Reconstructed computationally stained images shown in eFigure 1.1.b and 1.2.b (used for validation of the trained neural network) morphologically represented benign and malignant glands and stroma well enough to be consistently identified by pathologists (eFigure 1.1.c and 1.2.c) when compared with corresponding H&E dye stained images (eFigure 1.1.a and 1.2.a). A vast majority of tumor also showed annotator agreement. In some instances, "atypical" glands that were morphologically indeterminate for malignancy led to interpretative discrepancies however showed preserved morphology in the computationally stained images (e.g. arrows in eFigure 1.1.a and 1.1.b). Ground truth non-stained (eFigure 1.1.d and 1.2.d) and corresponding computationally destained images (eFigure 1.1.e and 1.2.e) are also shown for comparison. eFigure 1.4.b, 1.6.b and 1.7.b show the most reported areas of disagreement many of which are attributed to atypical glands that were hard to categorize on both images but were well represented on the computer-generated images (eFigure 1.4.c, 1.6.c and 1.7.c). eFigure 1.5 shows the uncommon Gleason pattern 5 tumors with comedo necrosis (eFigure 1.5.a, arrow). The morphology of the tumor glands is well maintained (eFigure 1.5.b, arrowheads), but the comedo necrosis is not visualized eFigure 1.5.b, arrow). The dye-stained image in eFigure 1.7.a contains an infrequently encountered scenario (indicated by an arrow), the presence of rare malignant glands that are not well visualized on the computationally stained image (eFigure 1.7.b, arrow). Despite this altered appearance, there was no impact on clinical diagnosis as the blinded reviewers scored these areas as tumor. Some glands are poorly formed on both the dye stained and the computationally stained image (eFigure 1.8.a, 1.8.b, arrows), leading to disagreement between raters, even though the computationally stained image were identical to dye stained image. Images shown in eFigure 1.9 presented a challenging labeling exercise where tumor cell cytoplasm was very pale and did not show significant contrast to the background stroma in the dye-stained image (eFigure 1.9.a). This cytoplasmic pallor was also well preserved in the computationally stained image (eFigure 1.9.b). Despite this, appearance of the nuclei and the slight difference in cytoplasmic texture made the tumor identifiable in both images (eFigure 1.9.c and 1.9.d). The computationally stained images shown in eFigure 1.10.b, 1.11.b, 1.13.b were well represented. Majority of the disagreement in these images arose due to tumor/non-tumor boundary and biopsy edge issues. Validation images in eFigure 1.11.b and 1.13.b illustrated additional high-quality examples of preserved morphology generated by the computationally staining algorithm, which confirmed accurate matching with dye stained images in benign conditions. Non-necrotizing granulomas, marked chronic inflammation, reactive stromal changes and proteinaceous debris were all morphologically identifiable in the computational stained images (eFigure 1.11.c). Pathologists unanimously scored the matched H&E dye stained and computationally stained images shown in eFigure 1.3 and 1.12 as benign.

© 2020 Rana A et al. *JAMA Network Open.*

eTable 5. Comparison of Tumor Grades Between Original Expert Microscopic Diagnosis

Comparison of tumor grades between original expert microscopic diagnosis [as reported in the Electronic Health Records (EHR)], using the Hematoxylin and Eosin (H&E) stained glass slide and the diagnosis of the computationally stained image. [*]Agreements confirmed upon re-review of the microscopic slide and additional supportive studies; [†] Not clinically significant within the context of the patients known tumor.

Image	Initial diagnosis after biopsy	Diagnosis using computationally stained image
1	40% grade 3 tumor in core	40% grade 3 tumor in core
2	50% grade 3 tumor in core	90% grade 3 tumor in core[*,†]
3	Benign core	Benign core
4	50% grade 3 tumor in core	50% grade 3 tumor (majority) with
		traces of grade 4 tumor[†]
5	50% grade 4 and 5 tumor on core	50% grade 4 and 5 tumor on core
	(G4 > G5)	(G4 < G5)[†]
6	40% grade 3 and 4 tumor in core	40% grade 3 and 4 tumor in core
	(G3 >> G4)	(G3 >> G4)[†]
7	40% grade 3 tumor in core	40% grade 3 and 4 tumor in core
		(G4 >> G3)[†]
8	40% grade 3 and 4 tumor in core	40% grade 3 and 4 tumor in core
9	20% grade 3 tumor in core	20% grade 3 tumor in core
10	90% grade 3 and 4 tumor in core	90% grade 3 and 4 tumor in core
11	Healthy core	Tiny focus of grade 3 tumor in core[*,†]
12	Healthy core	Healthy core
13	90% grade 4 tumor in core	90% grade 3 and 4 tumor in core
		(G3 << G4)[†]

© 2020 Rana A et al. *JAMA Network Open.*

eAppendix 5. Comparison With Patient Records

After expert re-review of the original slides and additional evaluation by immunohistochemistry, the original EHR diagnosis was overturned in two cases, resulting in two additional cases of agreement. Pathologists reviewing computer-generated core 11 were able to better identify the presence of rare glands of Gleason grade 3 tumors than those who had rendered the original EHR diagnosis of benign (eFigure 1.11 marked blue/green in the supplement). Microscopic re-review of the original glass slide confirmed that it indeed had a tiny focus of grade 3 tumor that was overlooked at the time of the original diagnosis. Subsequent immunohistochemical analysis revealed the absence of basal cells around the glands in question, confirming the diagnosis of carcinoma made during this study and revealing the diagnosis conferred on the computationally generated images to be correct. eFigure 1.2 was the only study biopsy that showed a significant difference in tumor fraction, as this study reported 50% tumor fraction and the original EHR report was 90%. Re-review of the original glass slide again showed this study fraction to be more accurate than the original diagnosis (eFigure 1.2). Otherwise, the tumor fraction identified in all the computationally generated images approximated the fraction reported in the EHR for all images as evident from eTable 5. None of the differences between EHR and computationally generated H&E diagnosis were clinically significant with regard to treatment decisions. A difference in grade of tumor was identified in a minor component of computationally stained images (eFigure 1.4, 1.7 and 1.13). The small foci of higher or lower grade tumor identified in computationally stained images (eFigure 1.4.c, 1.7.c, and 1.13.c), which were not reported at the time of original diagnosis, comprised a very small fraction of tumor volume. These were often associated with diagnostically indeterminate questions (e.g. whether a gland represented a rare focus of grade 4 tumor or if it was tangential sectioning of grade 3 tumor), and were not clinically significant in the context of the patient's known tumor at the time of original EHR reported diagnosis.

eReferences

1. Torr PH, Zisserman A. MLESAC: A new robust estimator with application to estimating image geometry. Computer vision and image understanding. Apr 1, 2000;78(1):138-56.
2. Lowe DG. Distinctive image features from scale-invariant keypoints. International journal of computer vision. Nov 1,2004;60(2):91-110.
3. Landis JR, Koch GG. The measurement of observer agreement for categorical data. Biometrics 33. 1977; 159–74.

© 2020 Rana A et al. *JAMA Network Open.*
